# Frequency of arboreality is correlated with longer hand skeletons in *Gorilla*: Analysis of a new skeletal sample of Bwindi mountain gorillas

**DOI:** 10.1111/joa.70121

**Published:** 2026-04-19

**Authors:** Elliot G. Greiner, Christopher B. Ruff, Charlotte King, Aventino Nkwasibwe, Thadee Muhire, Emmanuel Tibenda, Jason S. Massey, Juho‐Antti Junno, Rowan M. Sherwood, Jean Bosco Noheri, Fred Nizeyimana, Tierra Smiley Evans, Richard Muvunyi, Dennis Babaasa, Martha M. Robbins, Shannon C. McFarlin, Tracy L. Kivell

**Affiliations:** ^1^ Department of Human Origins Max Planck Institute for Evolutionary Anthropology Leipzig Germany; ^2^ Center for Functional Anatomy and Evolution Johns Hopkins University School of Medicine Baltimore Maryland USA; ^3^ Institute of Tropical Forest Conservation Mbarara University of Science and Technology Mbarara Uganda; ^4^ Dian Fossey Gorilla Fund Kinigi Rwanda; ^5^ Faculty of Medicine, Nursing, and Health Sciences Monash University Melbourne Australia; ^6^ Archeology University of Oulu Oulu Finland; ^7^ Department of Anthropology University of Michigan Ann Arbor Michigan USA; ^8^ Gorilla Doctors (Mountain Gorilla Veterinary Project, Inc.) Musanze Rwanda; ^9^ Gorilla Doctors (Mountain Gorilla Veterinary Project, Inc.) Davis California USA; ^10^ Gorilla Doctors (Mountain Gorilla Veterinary Project, Inc.) Kampala Uganda; ^11^ Department of Integrative Biology University of California Berkeley California USA; ^12^ Rwanda Development Board Kigali Rwanda; ^13^ Department of Primate Behavior and Evolution Max Planck Institute for Evolutionary Anthropology Leipzig Germany; ^14^ Department of Anthropology The George Washington University Washington DC USA

**Keywords:** African apes, climbing, ecomorphology, locomotion, metacarpal, phalanx

## Abstract

Understanding how ecological variation shapes skeletal morphology is important for linking observed locomotor behavior to anatomical correlates in extant hominoids, and for the subsequent interpretation of locomotor behavior in extinct taxa. In this study, we investigate ecomorphological variation in the metacarpals and phalanges across all five manual rays within *Gorilla*, focusing particularly on differences between western lowland gorilla (*Gorilla gorilla gorilla*) and the Rwandan (Volcanoes National Park) and Ugandan (Bwindi Impenetrable National Park) populations of mountain gorilla (*Gorilla beringei beringei*). This work incorporates the first substantial set of skeletal measurements from the Bwindi mountain gorilla population, derived from individuals recovered by the Mountain Gorilla Skeletal Project between 2018 and 2025. By comparing these data with samples from low and high‐elevation Grauer's gorillas (*Gorilla beringei graueri*), we assess how variation in arboreality correlates with digit element lengths, demonstrating that the Bwindi mountain gorillas exhibit intermediate metacarpal and phalangeal lengths between the more arboreal western lowland gorillas and the more terrestrial Virunga mountain gorillas. These findings indicate a correlation between longer digital rays in more arboreal populations, even within the same species, which may enhance grasping and stability on arboreal substrates. We show a population‐specific relationship between ecology and hand morphology in *Gorilla* and emphasize the value of documenting localized skeletal responses to environmental and behavioral variation to better interpret patterns in the hominoid fossil record.

## Graphical abstract

Variation in arboreality across gorilla populations is associated with systematic differences in manual digital lengths. Using a new dataset of linear metrics from the Bwindi mountain gorillas, we quantify metacarpal and phalangeal lengths in eastern and western gorilla populations across all five rays. Consistent with quantified behavioral differences, western lowland gorillas are the most arboreal and exhibit the longest manual digital elements, whereas Virunga mountain gorillas are the most terrestrial and show the shortest digits. Bwindi mountain gorillas are behaviorally intermediate in arboreality and display correspondingly intermediate digital lengths. These results demonstrate that localized ecological variation shapes hand proportions within *Gorilla*, providing a refined framework for interpreting locomotor adaptations in fossil hominoids.

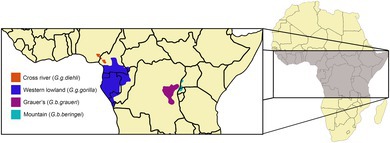

## 1 INTRODUCTION

Understanding the ecomorphological skeletal plasticity of mammalian groups is critical for interpreting their ecology in the deep past (Barr, 2018). While phylogeny and genetic drift significantly influence skeletal development and structure, the ecological factors moderating behaviors such as locomotion can also impact skeletal morphology at the population and individual levels (Arias‐Martorell et al., 2021; Cotter et al., 2009; Jashashvili et al., 2015; Lister, 2014; Lukova et al., 2024). However, because most ecomorphological research has broadly centered on interspecific differences (Foister & Felice, 2021; Jabbour & Pearman, 2016; Taylor & Slice, 2005), “localized” ecomorphological differences in the mammalian skeleton remain poorly understood, impacting interpretations of ecomorphological signals in the fossil record (Amson & Bibi, 2021; Barr, 2018; Dagher & Greiner, 2024; Marcé‐Nogué et al., 2017; Parins‐Fukuchi et al., 2019).

Within palaeoanthropology, the relationship between environment and mammalian bone shape has been used to reconstruct hominoid, including hominin, paleoenvironments (Barr, 2014, 2015; Kappelman et al., 1997), model climatic change (Barr, 2017; Fernandes, 2021; Greiner, 2024; Greiner et al., 2024; McGuire & Lauer, 2020), and directly evaluate hominin locomotor ecology itself (Almécija et al., 2010, 2015; Andrews et al., 1997; Hunt, 2016). Additionally, considerable research on interspecific variation in the ecomorphology of hominoid postcrania has been carried out and used to model the range of locomotor repertoires in extinct hominoid species (e.g., Almécija et al., 2021; Begun, 1992; Crompton, 2016; Hunt, 2016; Kivell et al., 2013, 2016; MacLatchy et al., 2000). However, to better contextualize the spectrum of environment‐specific locomotor patterns in extinct hominoids, it is also necessary to investigate the ecomorphological variation within and between extant hominoid populations.

Here, we investigate the ecomorphology of the hand skeleton (metacarpals and phalanges) in *Gorilla*, a phylogenetically closely related clade that lives within notably different ecological settings, through an analysis encompassing all five manual rays, expanding on the earlier work of Ruff et al. (2022). In particular, we focus on the potential morphological variation between the two extant populations of mountain gorillas (*Gorilla beringei beringei*): those of the Virunga Massif of Rwanda (Volcanoes National Park), Uganda (Mgahinga National Park) and Democratic Republic of Congo (Virunga National Park), in comparison to those of the Bwindi Impenetrable National Park, Uganda. The Bwindi mountain gorilla data represent the first robust sample of skeletal measurements from this population, derived largely from recently recovered skeletons. Our goal is to document how digital elements potentially respond to different ecological selective pressures between and within gorilla species, with a focus on variation in the frequency of arboreality. By clarifying the morphological response to differences in locomotor behavior and physical environments, these data will have implications for reconstructing the paleoecology of hominoids.

### 1.1 The hominid hand

Compared with other catarrhines, the hands of extant great apes are considered highly derived for grasping during a variety of orthograde arboreal behaviors, including long, curved fingers combined with a short thumb (e.g., Almécija et al., 2015; Prang et al., 2021; reviewed in Kivell et al., 2023). While African apes engage primarily in terrestrial knuckle‐walking (both on terrestrial and arboreal substrates), they also use climbing, suspension and (assisted) bipedalism at variable frequencies depending on the population (Hunt, 1992; Doran, 1997; Doran & Hunt, 1994; Drummond‐Clarke et al., 2022; King et al., 2025; Remis, 1995; Sarringhaus et al., 2014). Compared with *Pan*, *Gorilla* has relatively shorter fingers that are more similar in length across the rays; morphology that is most often linked to their greater terrestriality, larger body mass, and specific knuckle‐walking hand postures (Doran, 1997; Inouye, 1992; Prang et al., 2021). Recent morphological and phylogenetic comparative approaches suggest that *Pan* and *Pongo* are convergently derived in their hand (and upper limb) morphology, while *Gorilla*, with more similar intrinsic hand proportions to those of humans, represent a plesiomorphic morphotype (Almécija et al., 2015; Prang et al., 2021).

Differences in skeletal morphology between and within gorilla (sub)species have been shown to correlate with variation in the frequency of arboreality (Dunn et al., 2014; Inouye, 1992; Jabbour & Pearman, 2016; Ruff et al., 2018, 2022; Tocheri et al., 2011). Recent studies of gorilla behavior, habitat, and morphology provide opportunities for more nuanced examination of these relationships. While mountain gorillas of the Virunga Massif are indeed highly terrestrial, they represent a terrestrial extreme compared with other, more arboreal gorilla populations, including the Bwindi mountain gorillas (Doran, 1996, 1997; Robbins et al., 2025). Below we briefly review our current knowledge of the genetic relationships among extant gorillas, their ecology, and morphological differences.

### 1.2 Genetic relationships of *Gorilla*

**FIGURE 1:**
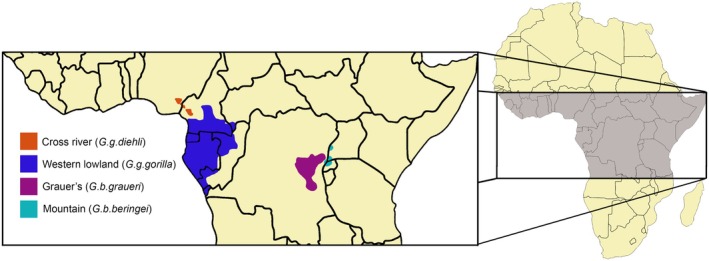
Extant distribution of western and eastern gorilla subspecies.

*Gorilla* is composed of two species, each with two subspecies: *Gorilla beringei* (eastern), comprising *G. beringei beringei* (mountain gorillas) and *G. beringei graueri* (Grauer's gorilla), and *G. gorilla* (western), comprising *G. gorilla diehli* (Cross River gorillas) and *G. gorilla gorilla* (western lowland gorillas) (Figure 1) (Xue et al., 2015). Eastern and western gorillas are estimated to have split between 1.2 and 3.0 million years ago (Ma), although these populations may have variably interbred until approximately 14,000–10,000 years ago (Städele et al., 2022; Thalmann et al., 2007; van der Valk et al., 2024; Xue et al., 2015). Mountain gorillas likely split from Grauer's gorillas, their monophyletic sister group, around the onset of the African Humid Period (~14,500 years ago), as newly interconnected forests allowed for a westward expansion of gorillas from the Virunga region into the area now occupied by Grauer's gorillas (van der Valk et al., 2024; Xue et al., 2015). The Bwindi and Virunga populations initially diverged during the Last Glacial Maximum (26,000–20,000 years ago), in part due to diminishing lowland rainforest habitat across eastern Africa (Roy et al., 2014; Tocheri et al., 2016). However, van der Valk et al. (2024) report a younger divergence time for Grauer's/Virunga mountain gorilla (12,000 years ago) than for Grauer's/Bwindi mountain gorilla (18,000 years ago), with gene flow subsequent to the divergence of Virunga and Grauer's populations, meaning that mountain gorillas form a paraphyletic clade.

### 1.3 Habitat, ecology and substrate use

Gorillas live across equatorial Africa in habitats ranging from dense, tropical lowland forests in western Africa to high‐elevation afromontane forests in eastern Africa (Robbins, 2011; Robbins & Robbins, 2018), and display diverse ecological behaviors moderated by food resource distribution and abundance within their habitats (Seiler et al., 2018; Seiler & Robbins, 2020) (Table 1). For western gorillas (excluding the Cross River subspecies, which is not evaluated in this study), this includes dense, lowland (<900 m) evergreen rainforests, as well as semi‐deciduous forests, with food resources that are more temporally and spatially variable than those of mountain gorillas (Gatti et al., 2004; Haurez et al., 2016; Head et al., 2011; Masi et al., 2009; Remis, 1997; Robbins et al., 2022). For eastern mountain gorillas, habitats range from lower altitude tropical forest to high altitude (~1160–2600 m) afromontane forest in Bwindi (McNeilage et al., 2001; Nkurunungi et al., 2004), to transitional lowland equatorial forests and high‐altitude dwarf montane forests in the Virunga Massif (~2300–4500 m) (Hall et al., 1998; Watts, 1984). Eastern Grauer's gorillas are found in tropical forest habitats at both high (~2100–2600 m) and low (<1300 m) elevations within the Democratic Republic of the Congo (Mehlman, 2008; van der Hoek, Pazo, et al., 2021).

**TABLE 1:** Breakdown of observed rates of arboreality (as % of total observation time) and elevation ranges for analyzed gorilla populations.

Population	Observed frequency of adult male (M) and female (F) arboreality	Habitat elevation
Western lowland gorilla (*G. g. gorilla*)	M = 20%, F = 35% (Loango, Gabon; Robbins et al., 2025)	<900 m
Low‐elevation Grauer's gorilla (*G*. *b. graueri*)	Not quantified	<1300 m
High‐elevation Grauer's gorilla (*G*. *b. graueri*)	Not quantified	~2100–2600 m
Bwindi mountain gorilla (*G. b. beringei*)	M = 18%, F = 21% (Robbins et al., 2025)	~1160–2600 m
Virunga mountain gorilla (*G. b. beringei*)	M = 2%, F = 7% (Doran, 1997)	~2300–4500 m

Seminal studies by Doran (1996, 1997) demonstrated that Virunga mountain gorillas are highly terrestrial, which was a pattern of substrate use often assumed to apply to all gorillas given limited data on other gorilla (sub)species or populations (e.g., Masi, 2004; Remis, 1995). However, recent observational (Robbins et al., 2025) and morphological (Harper et al., 2021; Jabbour & Pearman, 2016; Ruff et al., 2018, 2022; Tocheri et al., 2011) studies have incorporated information on variation in the frequency of arboreality across gorillas.

Adult male and female western lowland gorillas in Loango National Park, Gabon are arboreal on average 20% and 35% of total observed time, respectively (Robbins et al., 2025), and maintain relatively frugivorous diets (Doran‐Sheehy et al., 2009; Masi et al., 2015; Robbins et al., 2022, 2025). Though frugivory has been suggested as a driver of arboreal behavior in other primates, the absence of frugivory is not a strong predictor of terrestriality, and frugivory cannot be treated as a single causal factor; on its own, it does not account for the observed arboreality in western lowland gorillas (Estrada & Marshall, 2024; Robbins et al., 2025) (Table 1). Among eastern gorillas, frugivory has also been suggested to relate to differences in home range size and arboreality between high and low‐elevation Grauer's gorillas (Dunn et al., 2014; van der Hoek, Binyinyi, et al., 2021), although rates of arboreality have not been quantified for these populations. However, based on the groups that have been observed, both high‐ and low‐elevation populations are thought to have a frequency of arboreality that is intermediate between western lowland and Virunga mountain gorillas (Remis, 1995; Ruff et al., 2022).

Importantly, both populations of mountain gorillas also differ significantly in their frequency of arboreality. In the more frugivorous Bwindi mountain gorillas, adult females spend 21% and adult males spend 18% of total observational time engaged in arboreality (Ostrofsky & Robbins, 2020; Robbins et al., 2025). In contrast, among the Virunga mountain gorillas, with a primarily terrestrial herbaceous diet, adult females spend 7% and adult males 2% of their time arboreal (Doran, 1997). This is particularly relevant to this study because, although there is partial elevational overlap between the two regions (Bwindi's altitudinal range overlaps with ~33% of that of the Virunga Massif), the majority of the Virunga sample analyzed here is drawn from the Karisoke study area, which is a distinct, high‐altitude afromontane forest (2700–3400 m) that represents an ecological extreme for gorillas and which is characterized by a general scarcity of fruiting trees (Akayezu et al., 2019; Eckardt et al., 2019; Robbins et al., 2011, 2025). In contrast, the lower elevations in Bwindi support greater densities of key fruiting species.

Thus, based on current data, there is a large spectrum of variation in the frequency of arboreality among adults of both species and subspecies of gorillas, with western lowland gorillas being the most arboreal group and Virunga mountain gorillas the most terrestrial.

### 1.4 Morphological differences

Previous research has highlighted differences in postcranial morphology and proportions between eastern and western gorillas that have been linked to genetic, functional, or ecological differences (Carlson, 2005; Dunn et al., 2014; Groves, 1970; Harper et al., 2023; Inouye, 1992; Jabbour & Pearman, 2016; Prang & Tocheri, 2024; Ruff et al., 2018, 2022; Sarmiento, 1994; Schultz, 1930, 1951; Tocheri et al., 2011). Western gorillas have shorter trunks and longer limbs relative to body mass than Virunga mountain gorillas (Ruff et al., 2022). Western gorillas also show relatively greater forelimb bone strength than their eastern counterparts, potentially related to higher mechanical loads on the forelimb imposed by climbing (Ruff et al., 2018). More terrestrial eastern gorilla populations develop relatively stronger ulnae compared to their arboreal counterparts, potentially reflecting changes in hand position during more frequent bouts of quadrupedal knuckle‐walking (Ruff et al., 2018) and/or increased loading of the radius during climbing (Ruff et al., 2024). In contrast, populations purported to exhibit intermediate levels of arboreality, such as high‐elevation Grauer's gorillas, show forelimb strength proportions and long‐bone metrics that fall between those of western lowland and mountain gorillas (Ruff et al., 2018, 2022, 2024). More quantitative data on locomotor behavior for Grauer's gorillas are needed to further test these linkages between postcranial morphology and behavior in this subspecies, however.

Patterns of terrestriality and arboreality appear to correlate well with hand and foot proportions, which are generally absolutely and relatively longer in western gorillas than in eastern gorillas (Ruff et al., 2018, 2022). Though this observation has mainly been limited to measurements of the manual third ray (Inouye, 1992; Jabbour & Pearman, 2016; Ruff et al., 2018, 2022), longer hands and feet in general have been argued to be advantageous for increased arboreality in western gorillas compared with eastern gorillas; a longer digit length may be better to diffuse the loading incurred from power grips used on large supports during vertical climbing, as well as to allow the grasping of larger supports generally (King et al., 2025). Furthermore, Sarmiento et al. (1996) proposed that Bwindi mountain gorillas had relatively shorter hands (and feet) than their Virunga counterparts, but this was based on the assessment of one female Bwindi gorilla and thus has not been rigorously tested. These patterns have also been observed throughout ontogeny: Ruff et al. (2022) found that infant and juvenile western lowland gorillas have longer relative third metacarpal lengths than all other gorilla subspecies and populations. Though they did not find significant differences within subgroups of the eastern gorilla, they reported that Grauer's gorilla infants at both high and low elevations plotted toward the shorter end of the *eastern gorilla* distribution, reflecting relatively reduced bone lengths. While the differences in gorilla manual third ray proportions have been documented, it remains unclear whether and how arboreality may influence morphology across different rays of the hand, including the thumb, and what skeletal elements (e.g., metacarpals, proximal phalanges) contribute to these relative differences in length at the interspecific, intraspecific, and intrasubspecific levels.

## 2 PREDICTIONS

In line with previous work on the manual third ray (Inouye, 1992; Jabbour & Pearman, 2016; Ruff et al., 2022), we quantify the relative length of the hand rays (metacarpals, proximal and intermediate phalanges of rays 1–5) in western lowland, mountain (Bwindi and Virunga populations), and Grauer's (populations at both low and high elevations) gorillas to test for differences in hand proportions in relation to differences in the frequency of arboreality among these populations (Table 1). We predict that if hand proportions are driven by selective pressures favoring arboreal locomotion, western lowland gorillas will have proportionally longer rays for a given body mass or upper limb length than the eastern species. Furthermore, we predict that Bwindi gorillas will have relative ray lengths (i.e., relative to a body size proxy and relative to forelimb length) that are more similar to western lowland gorillas than to Virunga mountain gorillas, with the latter population having the shortest relative ray lengths within our sample. Within subspecies, we predict that if sex differences exist, females will have relatively longer ray elements than males, as females are observed to be more arboreal across gorilla populations (Remis, 1999; Robbins et al., 2025).

We predict that the relative length of the thumb will be more similar across gorilla populations than the fingers due to the fingers being typically considered more functionally important during arboreal grasping (Samuel et al., 2018; but see Neufuss et al., 2017) and the critical role of the thumb in food manipulation and processing (Napier, 1960; Neufuss et al., 2019).

Following Ruff et al. (2018, 2022), we hypothesize that gorilla populations will exhibit similar allometric slopes in hand bone scaling relative to body mass but differ in elevations (intercepts), with more arboreal populations (western lowland and Bwindi) showing relatively longer metacarpals and phalanges for a given body mass than more terrestrial populations.

Finally, although longer phalanges relative to metacarpals are associated with arboreal grasping (Susman, 1979), to better understand how variation in ray length is achieved, we explore how specific skeletal elements (i.e., metacarpals, proximal, and intermediate phalanges) vary among taxa and if the same relative patterns are found across all five rays.

## 3 METHODS

Excluding Bwindi specimens, adult *G. gorilla* and *Gorilla beringei* Mc3 and PP3 measurements were obtained from a previous study by Ruff et al. (2022), which included data from the following curatorial institutions: The Field Museum of Natural History (Chicago, USA), Museum of Comparative Zoology (Cambridge, USA), Swedish Museum of Natural History (Stockholm, Sweden), The National Museum of Natural History (Washington, D.C.), The Powell Cotton Museum (Birchington, UK), The Royal Belgian Institute of Natural Science (Brussels, Belgium), The Royal Museum for Central Africa (Tervuren, Belgium), and the Mountain Gorilla Skeletal Project (MGSP) in Rwanda (Volcanoes National Park). The present study expanded this dataset by collecting length data for the other four hand rays, using specimens from the National Museum of Natural History, the Royal Museum for Central Africa, and the MGSP. Where ever possible, specimens collected for the present study were from the same individuals as in Ruff et al. (2022) in order to analyze the variation among digits. In addition, novel Bwindi data for all five hand rays were collected from the Institute for Tropical Forest Conservation (ITFC; Ruhija, Uganda), which serves as the repository for Bwindi mountain gorilla specimens, including specimens collected since 2017 by the MGSP.

Mountain gorilla skeletons curated by the MGSP were recovered postmortem (or catalogued, for pre‐existing specimens) from Volcanoes National Park in Rwanda (from 2007 to 2025) and Bwindi Impenetrable National Park in Uganda (from 2017 to 2025), in collaboration with Rwanda Development Board, Uganda Wildlife Authority, Gorilla Doctors, and other partners (McFarlin et al., 2009). Most of these skeletons were well‐preserved, with relatively complete hand skeletons, and often were from known individuals (e.g., with documented age and sex). Individuals were considered adult if the epiphyses of all elements were fully fused.

Digital caliper measurements were collected for maximum proximodistal length for all available metacarpals, proximal and intermediate phalanges of the five hand rays, following Ruff et al. (2022) (Table 2). All measurements were rounded to the tenth of a millimeter. Identification of the ray to which each phalanx belonged followed morphological descriptions in Susman (1979). For specimens curated by the MGSP, phalangeal ray associations were recorded at the time of skeletal excavation for those cases where hand skeletons were recovered with their anatomical associations in situ.

**TABLE 2:** Metacarpal, proximal and intermediate phalangeal sample for each gorilla population and sex.

Bone	*G. b. beringei*			*G. b. beringei*			*G. b. graueri*			*G. b. graueri*			*G. g. gorilla*		
	(Bwindi)			(Virunga)			(Low elevation)			(High elevation)					
	*N*	Male	Female	*N*	Male	Female	*N*	Male	Female	*N*	Male	Female	*N*	Male	Female
Metacarpal 1	**24**	15	9	**45**	21	24	**3**	1	2	**12**	7	5	**14**	8	6
Metacarpal 2	**23**	15	8	**46**	24	22	**2**	0	2	**12**	6	6	**12**	7	5
Metacarpal 3	**25**	15	10	**56**	29	27	**9**	6	3	**27**	17	10	**53**	26	27
Metacarpal 4	**24**	15	9	**46**	24	22	**3**	1	2	**12**	6	6	**14**	8	6
Metacarpal 5	**24**	15	9	**46**	22	23	**3**	1	2	**12**	7	5	**11**	6	5
Prox. Phalanx 1	**19**	13	6	**2**	1	1	**2**	0	2	**5**	2	3	**9**	4	5
Prox. Phalanx 2	**21**	13	8	**7**	2	5	**1**	0	1	**3**	1	2	**13**	7	6
Prox. Phalanx 3	**23**	14	9	**42**	23	19	**7**	4	3	**20**	12	8	**52**	28	24
Prox. Phalanx 4	**21**	14	7	**1**	1	0	**1**	0	1	**3**	1	2	**10**	5	5
Prox. Phalanx 5	**18**	10	8	**7**	4	3	**2**	0	2	**7**	3	4	**13**	8	5
Int. Phalanx 2	**13**	8	5	**6**	4	2	**1**	0	1	**1**	0	1	**11**	7	4
Int. Phalanx 3	**18**	11	7	**11**	8	3	**2**	0	2	**4**	1	3	**12**	8	4
Int. Phalanx 4	**13**	9	4	**1**	1	0	**1**	0	1	**1**	0	1	**9**	5	4
Int. Phalanx 5	**11**	7	4	**7**	4	3	**1**	0	1	**1**	0	1	**12**	8	4

*Note*: The bold values indicates the total sum in the *N* columns.

The maximum lengths of the humerus, radius, femur, and tibia were taken from Ruff et al. (2022) for all non‐Bwindi gorillas. However, due to limited equipment in the field, maximum lengths of these elements were measured for the Bwindi gorillas using a rigid 1 m steel rule placed on a level cement floor with a perpendicular, fixed vertical backstop to replicate an osteometric board. Bones were oriented against the vertical stop, with measurements taken parallel to the longitudinal axis of the shaft following Ruff et al. (2022). Specifically, humeral length was taken from the superior point of the humeral head to the most distal point of the trochlea/capitulum; radial length from the proximal rim of the radial head to the distal tip of the styloid process; femoral length from the superior surface of the femoral head to the most distal point of the condyles; and tibial length from the intercondylar eminence to the distal tip of the medial malleolus. Right sides were preferentially measured, but lefts were substituted when unavailable. These long‐bone measurements were used both directly in interlimb proportion analyses and as a second scaling baseline for metacarpal and phalangeal lengths.

Variation in relative ray lengths was evaluated among individual elements, as well as between cumulative metacarpal and phalangeal lengths for each ray. All absolute lengths were first standardized by body mass estimated using the gorilla‐specific equations in Ruff et al. (2022) based on linear articular dimensions as predictor variables (described in Ruff (2002, 2007)). In this study, the mediolateral articular breadth of the distal femur was used as the predictor variable, as, when compared to gorillas of known body masses, this was found to be the best estimator of body mass for individuals with fully fused epiphyses (Burgess et al., 2018). Analyses scaled to estimated body mass were conducted with sexes pooled, as body mass represents a whole‐body size proxy that incorporates sexual dimorphism in overall size. However, to investigate potential sex differences in ray/segment lengths, we scaled to combined humerus and radius length to account for known sex differences in limb proportions (Ruff et al., 2022) and to avoid conflating proportional variation in the hand with sexual dimorphism in forelimb length.

Reduced major axis (RMA) regressions for proximal phalanges and metacarpals were also generated to characterize how hand bone lengths scale with body size. For each population, the slope and elevation were estimated for metacarpals and proximal phalanges for all five rays, and predicted values were calculated over the full range of log‐transformed estimated body masses observed in each population.

Metacarpal (Mc1‐5), proximal phalangeal (PP1‐5), and intermediate phalangeal (IP2‐5) maximum lengths across the gorilla populations were first log‐transformed and analyzed using an RMA regression of log([*digital element length*]) on log(*body mass*), and then divided by sex and analyzed again using an RMA regression of log([*digital element length*]) on log(*humerus + radius lengths*), with residuals extracted to assess proportional differences independent of size. Sample sizes differed for each skeletal element and across populations, and therefore, we only ran analyses on samples with *n* ≥5 individuals. Our sample sizes for the IP2‐5 were limited, especially for Grauer's gorillas, and thus the combined ray length (Mc + PP + IP) was only assessed in Bwindi and Virunga mountain gorillas and western lowland gorillas. Sex‐specific analyses were only conducted on third ray elements due to sample size constraints. Residuals were then compared across populations using Kruskal–Wallis and pairwise Wilcoxon tests (Holm‐adjusted) (Holm, 1979).

To assess interpopulation variation in phalanx–metacarpal proportions, analyses included all proximal phalanges (PP1–5) paired with their corresponding metacarpals (Mc1–5). Intermediate phalanges were excluded due to insufficient sample sizes (*n* < 5 per population). Log‐transformed proximal phalanx lengths were regressed against log‐transformed metacarpal lengths using sex‐specific RMA regressions. Residuals from these models represent proportional deviations in phalanx length relative to the corresponding metacarpal. Analyses were limited to population–sex groups with at least five specimens per element to ensure stability of RMA slopes and residual estimates, and mean residuals with standard errors were visualized to compare proportional differences among populations.

All analyses were performed and visualized in R (v4.3.0; R Core Team, 2023) using smatr, ggplot2, and dplyr.

### 3.1 Measurement error assessment

Because metacarpal and phalangeal measurements were collected by multiple researchers (EG, TM, RS), including third metacarpal data previously published in Ruff et al. (2022), we conducted a calibration exercise to ensure consistency. Each researcher measured the third metacarpal length of at least 10 specimens included in Ruff et al. (2022) and compared their results to the published values. Differences between observer and published values were calculated, and one‐sample tests were used to assess whether mean differences deviated significantly from zero, thereby evaluating the presence of systematic offsets. No significant differences were found between observers and published Mc3 values (mean absolute difference = 0.09–1.18 mm; all *p* > 0.05; *n* = 12–28 per observer). Mean directional offsets were small (|mean| <0.25 mm) and non‐significant, indicating high measurement repeatability and negligible systematic bias (Table S1).

Additionally, because the field protocol for long‐bone measurements in Bwindi could have introduced small systematic and random errors relative to osteometric‐board measurements, we conducted sensitivity analyses restricted to the Bwindi subset. For each Bwindi individual, we applied simulated measurement errors to the raw humerus, radius, femur, and tibia lengths by: (1) adding fixed offsets of −5, −3, −1, +1, +3, +5, or +10 mm; (2) multiplying lengths by scalars of 0.99, 0.97, 0.95, or 0.90; or (3) adding a fixed +10 mm offset plus normally distributed noise (SD = 2 mm) to simulate board‐to‐floor placement error. These modified values were then used to re‐fit log10–log10 ANCOVA models for radius–humerus and tibia–femur scaling. In all cases, the population × size interaction *p*‐value remained >0.05 (ranges reported in Table S2), showing that the differences in limb scaling between Bwindi and other gorillas were not affected by plausible levels of measurement error.

## 4 RESULTS

### 4.1 Comparison of size‐corrected digit length (Mc + PP + IP)

**FIGURE 2:**
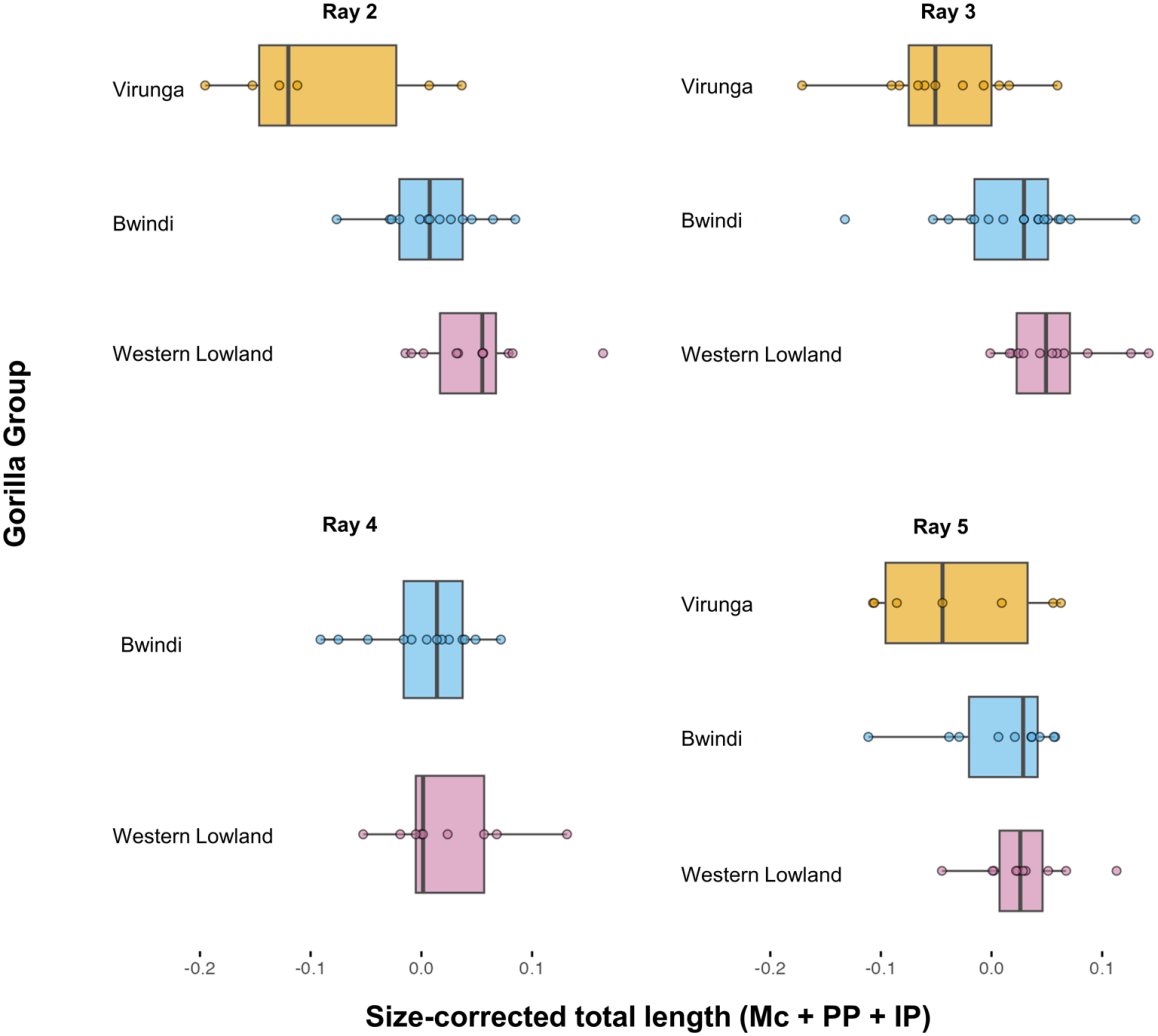
Variation across gorilla populations (pooled sexes) in size‐corrected total ray lengths across rays 2–5 (residuals from RMA regressions against body mass). Total ray length calculated as the sum of the maximum lengths of the respective metacarpal (Mc), proximal phalanx (PP), and intermediate phalanx (IP) for rays 2–5. Only bone × gorilla populations with ≥5 specimens are shown.

Residuals from RMA regressions of log‐transformed combined bone lengths on log‐transformed body mass (results only reported when ≥5 individuals were available) show that across gorilla populations, size‐corrected lengths for combined metacarpal, proximal, and intermediate phalanges for rays 2–5 vary systematically by known and, in the case of Grauer's gorillas, presumed frequencies of arboreality (Figure 2). Western lowland gorillas generally exhibit longer size‐corrected rays, while Virunga gorillas tend to have shorter relative lengths, and Bwindi gorillas fall intermediate to these populations. However, differences reach significance only in comparisons between Western and Virunga gorillas for rays 2 and 3, and between Bwindi and Virunga gorillas in ray 3 (Supporting Information S1; Table S3). This may be accounted for in part by the relatively small sample sizes for rays other 3, which is itself due to the smaller number of intermediate phalanges available (Table 1). For this reason, metacarpals and proximal phalanges are the focus for the remaining analyses.

### 4.2 Comparison of size‐corrected metacarpal and proximal phalanx lengths

**FIGURE 3:**
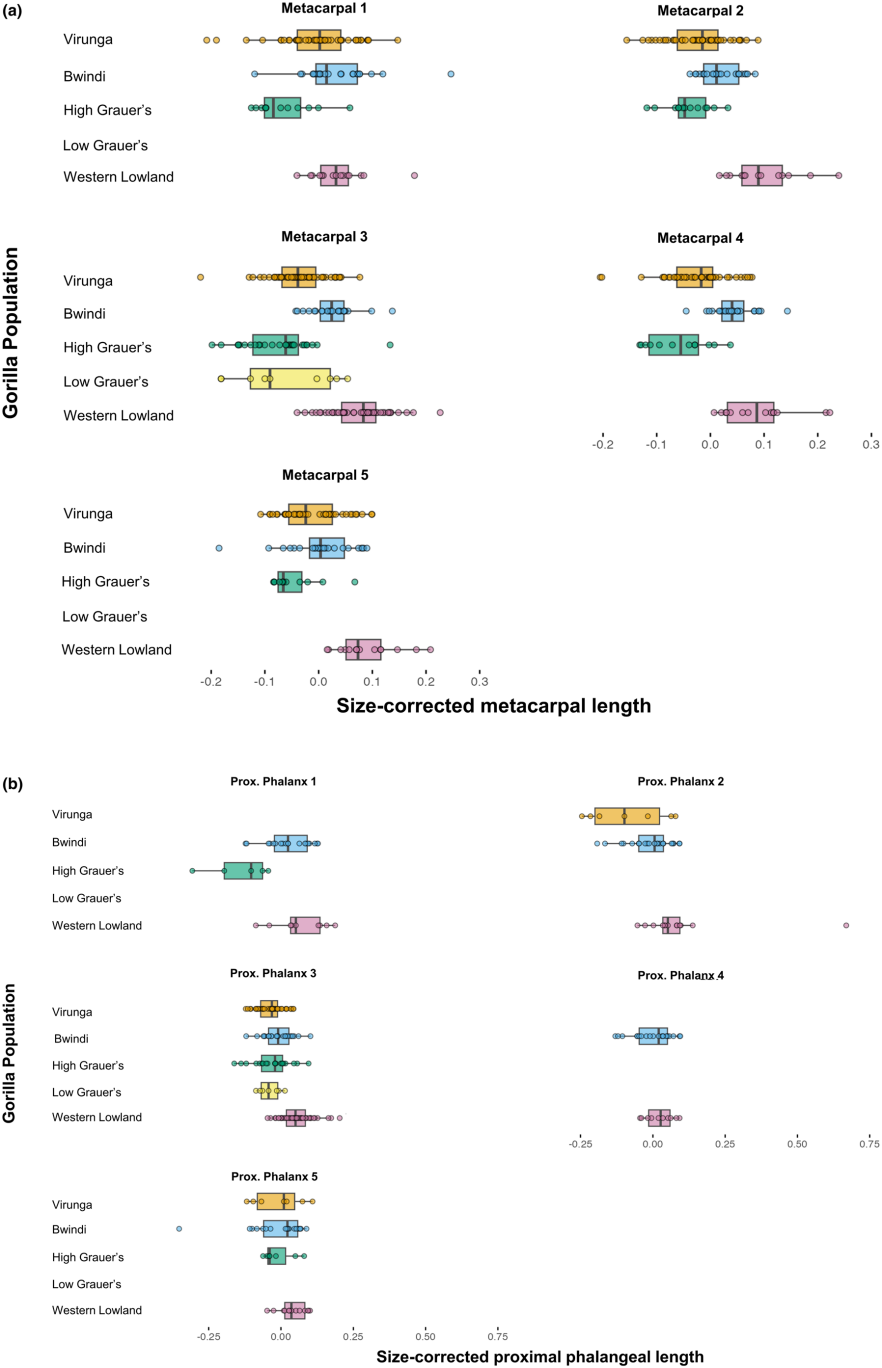
(a) Metacarpal length relative to body mass. Box and whisker plots of metacarpal length residuals derived from RMA regression of log(metacarpal length) on log(body mass). (b) Phalangeal length relative to body mass. Box and whisker plots of proximal phalangeal length residuals derived from RMA regression of log(phalangeal length) on log(body mass).

Figure 3 depicts results from residuals from RMA regressions of log‐transformed bone length on log‐transformed body mass that were used to separately assess relative Mc1–5 and proximal phalanx PP1–5 lengths across combined‐sex gorilla populations. Multiple significantly different relationships were found, with detailed results reported in Table 3.

**TABLE 3:** Summary of pairwise statistical comparisons among gorilla populations for metacarpal (Mc) and proximal phalanx (PP) lengths of each digital ray.

	Bwindi	Virunga	High G	Low G	Western low
Ray 1					
Bwindi			*B* **		
Virunga					
High G	*B* ***	V**			W**
Low G					
Western low			*W* *** (−)		
Ray 2					
Bwindi					W*
Virunga	*B* ** (***)				W*
High G	*B* ***				
Low G					
Western low	*W* *** (−)	*W* ***	*W* ***		
Ray 3					
Bwindi					*W* ***
Virunga	*B* ***				*W* ***
High G	*B* ***	V* (**)			*W* ***
Low G					*W* ***
Western low	*W* *** (−)	*W* ***	*W* ***	*W* *** (−)	
Ray 4					
Bwindi					
Virunga	*B* ***				
High G	*B* ***				
Low G					
Western low		*W* ***	*W* ***		
Ray 5					
Bwindi					
Virunga					
High G	B* (**)	V* (−)			
Low G					
Western low	*W* *** (−)	*W* ***	*W* ***		

*Note*: For each ray, metacarpal (Mc) results are shown below the diagonal and proximal phalanx (PP) results above the diagonal. Letter = group with longer element (residuals vs. body mass); Italics = also longer in line elevation tests; Parentheses = results differ when scaled to humerus + radius length, in level of significance, (−) = non‐significant or reverses in direction. Blank cells = non‐significant comparisons, all analyses. The black boxes demarcate the boundary between metapodials and phalanges.

Abbreviations: B, Bwindi; V, Virunga; W, western lowland gorillas.

* *p* < 0.05.

** *p* < 0.01.

*** *p* < 0.001.

Western lowland gorillas had significantly longer Mc2–Mc5 than high‐elevation Grauer's and Virunga gorillas (all *p* < 0.0001), and significantly longer Mc2‐Mc3 than Bwindi gorillas (*p* < 0.001). Bwindi gorillas were significantly longer than both Virunga and high‐elevation Grauer's gorillas in Mc2–Mc4 (all *p* < 0.01 and *p* < 0.001, respectively), while Virunga gorillas were also significantly longer than high‐elevation Grauer's in Mc2–Mc3 (*p* < 0.05). Mc1 showed greater overlap among populations, but high‐elevation Grauer's gorillas were significantly shorter than Bwindi (*p* < 0.001).

Fewer significant differences were observed among populations in the manual proximal phalanges than in the metacarpals, reflecting greater overlap and smaller sample sizes. In PP3, the best‐sampled phalangeal bone, western lowland gorillas were significantly longer than all other populations (all *p* < 0.001), whereas Bwindi did not differ significantly from Virunga or either Grauer's population. In PP1, Bwindi and western lowland gorillas did not differ, but Bwindi was significantly longer than high‐elevation Grauer's gorillas (*p* < 0.01). For PP2, Bwindi gorillas were shorter than western lowland gorillas (*p* < 0.05) but did not differ from Virunga (*p* = 0.208), while Virunga were also shorter than western lowland (*p* < 0.05). No significant differences were detected among populations for PP4 or PP5.

### 4.3 Metacarpal and proximal phalangeal length relative to body mass proxy

Regression analyses of log‐transformed maximum metacarpal and proximal phalanx lengths against log‐transformed body mass revealed approximately isometric to slightly negatively allometric scaling relationships across all sex‐pooled gorilla populations (RMA slopes = 0.19–0.32 (Mt) 0.11–0.30 (PP); isometric expectation = 0.333; Figure S1a; Figure S1b). The isometric expectation (0.333) represents theoretical length–mass scaling, and our slopes are consistent with previously reported negative allometry in gorilla limb bones (Ruff et al., 2022). In the metacarpals, population‐specific slopes did not differ significantly, whereas elevations did (all *p* < 0.0001; Table S4), indicating that although scaling patterns are broadly shared, there is consistent interpopulation variation in digital lengths for a given body size. Pairwise comparisons showed that Bwindi and western lowland gorillas had generally higher intercepts (longer metacarpals relative to body mass) than Virunga and high‐elevation Grauer's gorillas, with the strongest contrasts in Mc2–4. Mc5 showed fewer differences.

The Mc1 shows a weaker version of this pattern with fewer significant pairwise differences than metacarpals 2–5 (Figure 3a; Figure S1a). Bwindi gorillas have relatively longer Mc1 lengths than high‐elevation Grauer's and Virunga gorillas, while Virunga and western lowland gorillas are also longer than high‐elevation Grauer's gorillas (all *p* < 0.05). Thus, Mc1 largely follows the same trend but with reduced contrast among populations.

In the proximal phalanges, slope elevations differed significantly among populations for PP1–3 (all *p* ≤ 0.019; Figure S1b; Table S5). Pairwise elevation comparisons revealed that western lowland gorillas generally had significantly higher elevations (longer phalanges relative to body mass) compared to Virunga and high‐elevation Grauer's gorillas, with Bwindi gorillas falling intermediate between these two populations. Like the Mc5, PP4 and PP5 showed broad overlap among populations with no significant differences in relative length between populations.

### 4.4 Metacarpal and proximal phalangeal length relative to upper limb length

**FIGURE 4:**
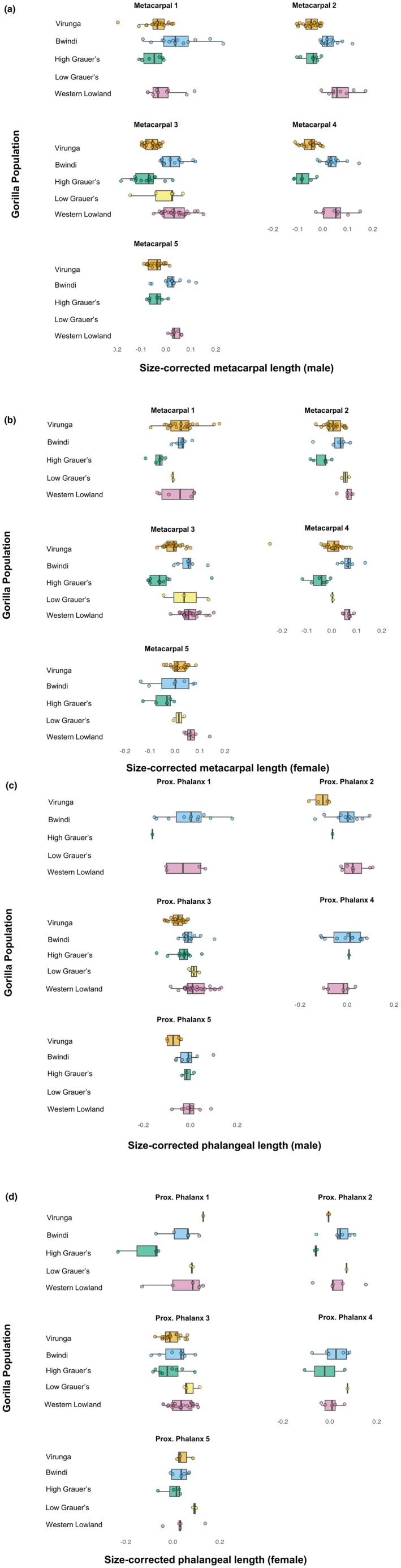
(a) Metacarpal length relative to humeral + radial length (male). Box and whisker plots of male metacarpal length residuals derived from RMA regression of log(metacarpal length) on log(humeral + radial length). (b) Metacarpal length relative to humeral + radial length (female). Box and whisker plots of female metacarpal length residuals derived from RMA regression of log(metacarpal length) on log(humeral + radial length). (c) Phalangeal length relative to humeral + radial length (male). Box and whisker plots of male phalangeal length residuals derived from RMA regression of log(phalangeal length) on log(humeral + radial length). (d) Phalangeal length relative to humeral + radial length (female). Box and whisker plots of female phalangeal length residuals derived from RMA regression of log(phalangeal length) on log(humeral + radial length).

To assess variation in hand bone proportions in females and males relative to upper limb size, we performed RMA regressions of log‐transformed metacarpals 1–5 and proximal phalanx 1–5 lengths against the log of combined humeral and radial lengths (Figure 4a–d; Tables S6–S9).

Figure 4a,b depict residuals from RMA regressions of log‐transformed metacarpal lengths on log‐transformed humerus + radius length used to assess relative Mc1–Mc5 lengths separately in male and female gorillas (Tables S6 and S7). Among males, western lowland gorillas had significantly longer Mc2–5 than both high‐elevation Grauer's and Virunga (all *p* ≤ 0.002) and longer Mc2–3 than Bwindi males (*p* ≤ 0.0005). Bwindi males were significantly longer than both Virunga and high‐elevation Grauer's males in Mc2–4 (*p* ≤ 0.005), while Virunga and high‐elevation Grauer's males did not differ significantly. Mc1 showed greater overlap, though Bwindi males retained slightly longer relative lengths than high‐elevation Grauer's (*p* = 0.046) and Virunga (*p* = 0.013).

Among females, western lowland gorillas again possessed the longest metacarpals, significantly exceeding high‐elevation Grauer's and Virunga females in Mc3–5 (all *p* < 0.05). Bwindi females were also longer than high‐elevation Grauer's and Virunga females in Mc3–4 (all *p* < 0.01), while Virunga females were longer than high‐elevation Grauer's females in Mc1–2 (all *p* < 0.05). Overall, the gradient of western lowland > Bwindi > Virunga remained consistent across sexes—with the greatest contrasts in Mc2–4.

Figure 4c,d depict residuals from RMA regressions of log‐transformed proximal phalanx (PP1–5) lengths on log‐transformed combined humeral and radial length used to separately assess relative phalangeal proportions in male and female gorillas (Tables S8 and S9). Significant interpopulation differences occurred only in the third proximal phalanx (PP3). In both sexes, Bwindi and western lowland gorillas exhibited significantly higher (longer) size‐corrected PP3 residuals than Virunga gorillas, while western lowland males also exceeded high‐elevation Grauer's (*p* < 0.01). For males, these contrasts were highly significant (western lowland > Virunga, *p* < 0.001; Bwindi > Virunga, *p* < 0.001), while for females only western gorilla females were significantly longer than Virunga females (western lowland > Virunga, *p* < 0.05).

### 4.5 Phalangeal/metacarpal proportions in third ray

**FIGURE 5:**
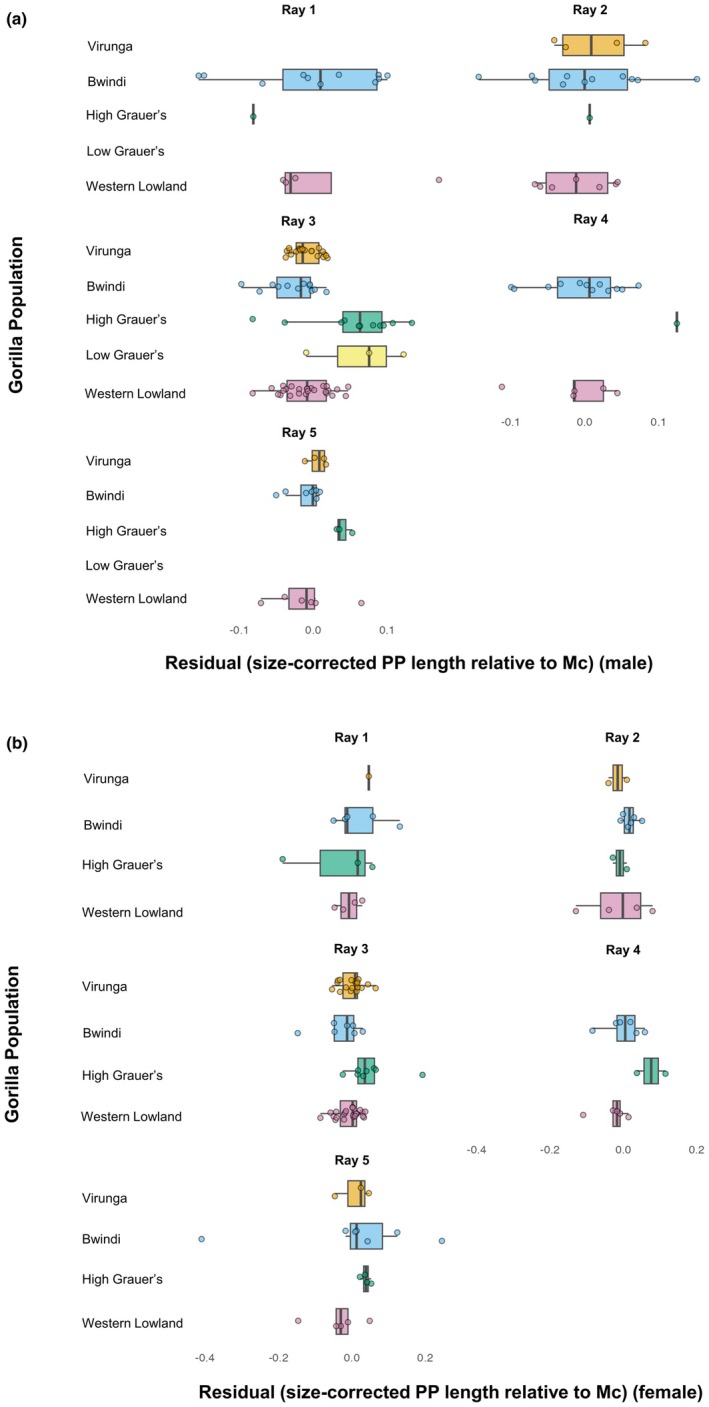
(a) Box and whisker plots of male proximal phalangeal length residuals (log[PP length]~log[MC length]) by population. (b) Box and whisker plots of female proximal phalangeal length residuals (log[PP length]~log[MC length]) by population.

Figure 5a,b show residuals from RMA regressions of log‐transformed proximal phalanx lengths on log‐transformed metacarpal lengths (PP3~Mc3), isolating phalanx–metacarpal proportions independently of upper limb size. Analyses were conducted separately by sex.

Among females, the PP3 exhibited significant population‐level differences (Kruskal–Wallis *p* < 0.05; Tables S10 and S11). Pairwise Wilcoxon tests (Holm‐adjusted) revealed that high‐elevation Grauer's females had significantly longer PP3s relative to Mc3 length than both Bwindi (*p* < 0.05) and Virunga (*p* < 0.05) females, while western lowland females did not differ significantly from either Bwindi or Virunga (*p* > 0.05).

Among males, the PP3 also showed an overall significant Kruskal–Wallis result (*p* = 0.004; Tables S10 and S11). Pairwise Wilcoxon tests (Holm‐adjusted) revealed that high‐elevation Grauer's males had significantly longer PP3s relative to Mc3 length than western lowland (*p* < 0.01), Bwindi (*p* < 0.05), and Virunga (*p* < 0.05) males. No other pairwise differences were significant after correction.

Mean residuals (Table S11) indicate that high‐elevation Grauer's individuals have proportionally longer PP3s relative to Mc3 length than all other populations, whereas Bwindi show the lowest residuals, consistent with proportionally shorter PP3s; Virunga and western lowland gorillas are intermediate. However, because several pairwise comparisons are non‐significant after correction, these relationships should be interpreted as trends rather than categorical differences.

## 5 DISCUSSION

Following on previous work demonstrating the correlation between hominid hand morphology and locomotor behavior (e.g., Marchi, 2005; Ruff et al., 2018, 2022; Susman, 1979; reviewed in Kivell et al., 2023), we tested several predictions regarding inter‐ and intra‐population variation in gorilla hand proportions relative to differences in the frequency of arboreality. This study presents the first extensive data set on the Bwindi mountain gorillas, who are significantly more arboreal than Virunga mountain gorillas (Doran, 1996, 1997; Robbins et al., 2025); thus allowing us to explore ecomorphology of the hand at the population level.

Our analyses support the hypothesis that the more arboreal western lowland gorillas possess relatively longer rays compared to Virunga and high‐elevation Grauer's gorillas. However, the position of Bwindi gorillas was more complex than expected: rather than consistently aligning closer to western lowland gorillas, as predicted, Bwindi gorillas were often intermediate between the western lowland and Virunga and Grauer's gorillas. Sexual dimorphism in ray proportions was not statistically significant within each population, and while the first ray showed less differentiation between populations than rays 2–5, subtle population‐level variation was still evident. Each of our predictions and results is discussed in more detail below. These findings support and extend earlier studies of gorilla limb proportions (Inouye, 1992; Jabbour & Pearman, 2016; Ruff et al., 2018, 2022), while the inclusion of Bwindi gorillas for the first time reveals a more nuanced pattern of variation within *Gorilla* than previously recognized.

### 5.1 Differences in metacarpal and phalangeal relative lengths

We predicted that western lowland and Bwindi gorillas, as the more arboreal populations, would have relatively longer metacarpals and phalanges compared to the more terrestrial Virunga population (Robbins et al., 2025; Figures 3 and 4). Overall, our analyses support prior work (Ruff et al., 2022) suggesting a correlation between relatively longer digital elements in more arboreal western lowland gorillas compared to all eastern gorillas. Furthermore, within the eastern gorilla sample, the Bwindi gorillas show relatively and absolutely longer metacarpals than the other eastern populations.

Western lowland gorillas generally exhibit the longest metacarpals and proximal phalanges relative to body size and to upper limb length, consistent with their more frequent arboreal behavior and the functional demands of grasping and climbing. Virunga gorillas, by contrast, had the shortest relative metacarpals and proximal phalanges, reflecting their documented (Doran, 1996, 1997; Robbins et al., 2025) or presumed more terrestrial locomotor repertoire. Bwindi gorillas consistently fell in an intermediate position: for some elements (e.g., Mc2, Mc4 and PP1) they overlapped in relative lengths with western lowland gorillas, but in others (e.g., PP3) they were closer to Virunga and Grauer's populations. PP5, like Mc5, showed broad overlap among populations with no consistent differentiation. However, when the intermediate phalanges are included within the digit length, Bwindi is more similar to western lowland gorillas than to Virunga gorillas in rays 2–5. This pattern indicates that more arboreal Bwindi gorillas generally have longer finger lengths than the more terrestrial Virunga gorillas, but their longer fingers may be developmentally achieved in a different way from that of arboreal lowland gorillas. Bwindi gorillas have longer metacarpals, like lowland gorillas, but may increase finger length via longer intermediate, rather than proximal, phalanges.

Functionally, elongation of the central rays (Mc2–4) would enhance grasping efficiency during arboreal locomotion by increasing palmar contact area and stability on arboreal supports. When travelling arboreally, Loango western lowland gorillas and Bwindi mountain gorillas most commonly use diagonal hand grips during locomotion on small supports (~10 cm in diameter) and power grips for stabilization on large supports (typically >10 cm in diameter); these hand postures place the metacarpals in direct contact with the support (King et al., 2025; Neufuss et al., 2017). Relatively longer metacarpal lengths could subsequently stem from selection to increase the size of the palm, allowing for more stable arboreal locomotion along the large supports required for the large body mass of gorillas, and perhaps compensate for the lack of strongly curved phalanges (Susman & Stern, 1979). Additionally, the stronger signal for metacarpal lengthening seen in males could relate to a need for them to adapt climbing strategies to accommodate their large body masses to remain stable on tree branches, as falls from trees or other high points can lead to injury or death (Hoffman, 2012; Neufuss et al., 2018).

In contrast to the central rays, Mc5 shows a weaker and less consistent association with arboreality across gorilla populations, which may reflect the fact that the fifth ray is strongly involved in several locomotor and manipulative behaviors. Kinetic data indicate that, relative to chimpanzees, gorillas more frequently load all four fingers during knuckle‐walking (Matarazzo, 2013); a pattern consistent with their more similar ray lengths that contributes to a broader and more stable weight‐bearing platform during terrestrial locomotion (Inouye, 1992). They also employ a high degree of ulnar deviation at the wrist during arboreal climbing and descent, which likely increases functional involvement of the ulnar side of the hand (King et al., 2025; Neufuss et al., 2017). In addition, the fifth digit contributes to a wide range of manipulative and food‐processing grips that involve all fingers and opposition with the thumb (Neufuss et al., 2019). Together, these multiple functional roles may impose relatively similar selective pressures on Mc5 across gorilla populations, reducing the degree to which variation in arboreality is reflected in its length, in a manner analogous to the thumb. We emphasize that detailed in vivo kinematic and kinetic data for individual hand rays remain sparse and that further biomechanical studies, both in the zoo and, especially, natural settings are needed to evaluate these hypotheses.

However, in addition to locomotor differences, climatic variation might also contribute to interpopulation differences in digital proportions, consistent with Allen's Rule (Cardini et al., 2007; Strasser, 1992). Extremity length relative to body size is known to covary with ambient temperature in primates and other mammals, generally showing relative shortening in colder environments (Ruff, 1994; Ruff et al., 2022). Ruff et al. (2022) found that distal limb segments are relatively shorter in mountain and Grauer's gorillas than in western lowland gorillas, covarying with colder conditions at higher elevations, but also noted that locomotor behavior strongly covaries with these same environmental gradients. They argued that while climate likely contributes to extremity shortening, functional demands associated with terrestriality exert a stronger and more direct influence, particularly on digital proportions. Moreover, the relatively conserved phalangeal proportions they found across gorilla populations may reflect functional canalization of grasping elements against climatic effects, suggesting that locomotor function exerts stronger selection on these elements, while metacarpal variation may reflect both climatic and locomotor influences.

A second consideration concerns the relative roles of genetic inheritance and developmental plasticity in shaping adult digital proportions. Ruff et al. (2022) found that interpopulation differences in gorilla hand and foot bone lengths were already present in infants, implying a strong genetic component to these traits. They also reported little evidence for environmentally induced gradients across all eastern gorillas when lengths were scaled to body mass, consistent with canalization of hand and foot proportions inherited from a more terrestrial ancestor. However, subtle environmental effects were apparent in intra‐limb proportions—metacarpal, metatarsal, and phalangeal lengths relative to combined humeral and radial length—suggesting limited developmental responses to local conditions such as temperature. In this study, metacarpals show a clearer interpopulation gradient than proximal phalanges, particularly among the Virunga, Bwindi, and western lowland populations, consistent with either greater developmental plasticity or stronger recent selection on metacarpal proportions. Phalangeal lengths, in contrast, vary mainly between eastern and western populations, consistent with greater genetic canalization linked to their locomotor importance. Thus, the observed patterns may reflect a combination of developmentally mediated climatic effects on metacarpals and genetically mediated locomotor constraints on phalanges, with different degrees of influence across elements and rays.

#### 5.1.1 Relative length of the thumb

We also found a weaker association between length and arboreality in the Mc1 and PP1 across our gorilla populations. Bwindi gorillas generally had longer Mc1 and PP1 absolute lengths in the sample; however, when corrected by either body mass or upper limb length, there was broad overlap between all populations in the sample, with only high‐elevation Grauer's significantly differing from the other populations in having the shortest Mc1 lengths.

While the first ray is critical for gripping arboreal supports across both sexes and populations, during the climbing of large diameter supports it is most commonly held in adduction to the other digits (i.e., not used in opposition to them; Neufuss et al., 2017). Diagonal power grips, where the thumb is used in opposition, occur less frequently, as they are mainly utilized for climbing on medium‐sized supports that enable the gorilla to wrap the thumb around it (King et al., 2025; Neufuss et al., 2017). Additionally, since the first ray does not bear load during knuckle‐walking, it might also be able to maintain certain proportions for dexterity and forceful opposition during other non‐locomotor behaviors, such as food processing (e.g., stem peeling, leaf removal, bark stripping, fruit plucking), grooming, or carrying objects or infants (King et al., 2025; Napier, 1960; Neufuss, 2017; Saito et al., 2021). Subsequently, the morphological balance in the thumb of the functional demands of grasping during manipulation and locomotion may complicate the generalized pattern of digital lengthening seen the central rays (Mc2–Mc4) related to locomotor demands.

### 5.2 Allometry

We predicted, based on previous research (Ruff et al., 2022), that more arboreal populations are expected to have relatively longer metacarpals and phalanges for a given body mass (i.e., higher elevation) compared to more terrestrial populations, but that scaling to estimated body mass (i.e., allometric slope) would be similar across populations. Our predictions were supported. Building on the analysis of the third ray in Ruff et al. (2022), we showed similar scaling across our gorilla populations in rays 2–5. Moreover, more arboreal western lowland and Bwindi gorillas had significantly higher elevations than more terrestrial Virunga and Grauer's gorillas, indicating that for any given body mass they possess proportionally longer metacarpals and phalanges, consistent with results of the RMA residual analyses.

The conservation of slope values across populations suggests that developmental and biomechanical constraints on digital scaling are broadly shared within *Gorilla*, whereas elevation differences potentially reflect population‐level ecological or behavioral specialization. In other words, while all gorillas follow the same overall scaling trajectory, arboreal populations maintain relatively elongated digits, likely as an adaptive response to increased grasping demands during climbing and suspensory behaviors.

### 5.3 Sex differences

We predicted that if sex differences existed, female gorillas would exhibit relatively longer rays than males given that females have been shown to be more arboreal within most gorilla populations (Doran, 1997; Robbins et al., 2025), as well as in other apes (e.g., Doran, 1993; Drummond‐Clarke et al., 2022; Thorpe & Crompton, 2005). We found no significant differences between females and males within any of our gorilla populations (Figure S1), suggesting that selection for digital lengths is preserved between sexes. While males are less arboreal, they may require relatively longer fingers to grasp wider arboreal supports due to their larger body size compared with females (males are generally 150–210 kg compared to 70–100 kg for females) (Ruff et al., 2022; Smith & Jungers, 1997; Zihlman & McFarland, 2000).

However, while hand postures have not been well‐studied across gorilla populations, in the Loango population, where it has been quantified, palmigrady remains the most common hand posture during arboreal locomotion across the sexes and all age groups (King et al., 2025). Thus, this palmigrade hand posture potentially reduces selective pressure for sex‐specific differences in digital proportions by providing a stable, broadly shared locomotor strategy (King et al., 2025). These results suggest that, despite sex‐specific ecological differences in arboreal reliance, overall hand proportions are strongly conserved between sexes within populations, likely due to the shared role they have in arboreal stabilization between males and females.

### 5.4 Metacarpal/phalangeal proportions

Although longer phalanges relative to metacarpals have been associated with arboreal grasping (Susman, 1979), to better understand how variation in ray length is achieved among gorillas, we investigated metacarpal/proximal phalangeal length proportions of the third ray in our samples. The interpopulation differences observed here do not represent the same degree of proportional divergence documented in interspecific comparisons of metacarpal and phalangeal lengths relative to body mass or total limb length, suggesting that population‐level variation of metacarpal/phalangeal proportions within gorillas is comparatively subtle, and that these patterns cannot be applied across the entire hand.

The slight differences in third ray proportions between the populations, particularly the high‐elevation Grauer's gorillas, could either represent proportions that are genetically “fixed” at the subspecies level, or reflect digital proportions arising from unique manual behaviors and/or arboreal hand postures. However, among the remaining populations proportional differences are less pronounced and not statistically distinct, which makes strong functional conclusions less convincing. Even so, some ecological interpretations remain plausible. In Virunga gorillas, shorter metacarpals could increase the stability of the wrist by bringing it closer to the ground, while a relatively longer proximal phalanx could further increase stability by permitting less multiaxial movement during terrestrial knuckle‐walking (Matarazzo, 2013). In contrast, the western lowland and, more dramatically, Bwindi, gorilla pattern of relatively longer third metacarpals in relation to the proximal phalanges could expand palmar contact across arboreal surfaces while still allowing for efficient terrestrial knuckle‐walking, representing a tradeoff for an opportunistically arboreal ape that needs to remain terrestrially stable (Byrne & Byrne, 1991; Drapeau, 2015).

However, even if these ecological influences are responsible for the broad differences observed here, they will not be the only influence on this pattern. The functional significance of the middle rays in both supporting weight and contributing to manual dexterity (Byrne & Byrne, 1991; Napier, 1960; Susman, 1979; Watts, 1984) also likely factors into these differences. In order to better understand the potential selective pressures of these behaviors on metacarpal and phalangeal proportions within and between gorilla populations, the functional trade‐offs of locomotion and dexterous behaviors (e.g., food‐processing strategies) should be further considered in the context of other morphological features, such as digital curvature (Neufuss et al., 2019).

### 5.5 Evolutionary and biogeographic interpretation

Climatic cooling and aridification during the Last Glacial Maximum (ca. 26–20 ka) are inferred to have reduced and fragmented forest habitats, likely increasing isolation among eastern gorilla populations, whereas postglacial warming and humid conditions promoted forest expansion and renewed connectivity in central African forests between ~13 and 5 ka (Dunn et al., 2014). Genomic analyses suggest that divergence among eastern gorilla lineages and episodes of post‐divergence gene flow occurred largely during the late Pleistocene, with evidence for admixture events persisting into the terminal Pleistocene and early Holocene (van der Valk et al., 2024). In this context, traits that have a strong and consistent ecological signal, such as elongation of the central hand rays, may reflect selection related to arboreal locomotion across populations, whereas traits showing weaker or inconsistent signals may be more strongly constrained and/or periodically homogenized during intervals of renewed connectivity.

This pattern suggests that allopatric divergence among gorilla populations may have played an important role in shaping adaptive responses to arboreal lifeways, particularly when considered alongside other developmental, behavioral, and physiological differences documented between the Bwindi and Virunga mountain gorilla populations (Robbins et al., 2023; Robbins & Robbins, 2018; Stanford, 2001). These include differences in interbirth intervals, ages at weaning and natal dispersal, as well as population‐specific gene variants across all gorilla subspecies associated with muscular development and hair morphology tentatively interpreted as reflecting local adaptation (Robbins et al., 2023; Robbins & Robbins, 2021; van der Valk et al., 2024). Taken together, these differences indicate that morphological and physiological responses to arboreal locomotion and climate may have evolved over relatively short timescales following population isolation. Although the present study focuses on digital elements because of their paleontological relevance, the correspondence of morphology in general with broader patterns of physiological and genomic differentiation underscores the importance of integrating skeletal, ecological, and genomic perspectives when interpreting adaptive variation in both extant and fossil hominoids.

## 6 CONCLUSIONS

Western lowland gorillas display longer relative metacarpal and phalangeal lengths across hand rays 2–5 than the more terrestrial Grauer's and Virunga gorillas, with the metacarpal lengths of the Bwindi mountain gorillas falling in between western and eastern gorilla populations. Taken together, we propose that the relatively longer metacarpals of the more arboreal western lowland and Bwindi mountain gorillas developed as a consequence of increased surface contact during arboreal locomotion to mitigate risks of falling given their large body sizes, as well as to provide a larger contact point for propulsive force and braking during vertical climbing. In contrast, we argue that the relatively shorter third metacarpals of the more terrestrial Virunga mountain gorillas reduce the distance of the wrist from the ground during terrestrial knuckle‐walking. The first ray, by comparison, may be shaped by a broader functional compromise between locomotor stabilization and manipulative demands, which may explain its weaker ecological signal across populations.

An alternative explanation is that population history (e.g., drift following bottlenecks) contributed to among‐population differences in hand proportions within and between the eastern and western species. However, two observations argue against drift as a sole cause: (1) The most pronounced population differences are evident in metacarpals 2–4, which are functionally tied to arboreal support use; (2) allometric slopes are conserved while elevations differ. Nonetheless, due to our evolving understanding of genetic structure within and between eastern gorilla populations (van der Valk et al., 2024), we cannot completely exclude the influence of demographic effects and population history on observed length differences.

Future research should evaluate if these differences are mirrored in cortical thickness of the digital rays, as well as expand the metacarpal and phalangeal data to include curvature. Additionally, breadth and strength data from the metacarpal and phalangeal shafts would likely help elucidate locomotor patterns in the hand rays, as maximum length metrics alone do not reflect mechanical loadings of the elements. Integration of GPS‐based movement ecology or 3D hand kinematics from wild populations might also provide stronger behavioral correlations for observed morphological trends.

Ultimately, this study demonstrates the close relationship between ecology and skeletal form, underscoring the need to characterize localized environmental responses of the hominoid skeleton to better inform morphological variability in the fossil record.

## AUTHOR CONTRIBUTIONS

Conceptualization: EGG, TLK, CBR. Skeletal Collection and Curation: SCM, AN, ET, TM, JBN, TSE, FN, JSM, J‐AJ, EGG, DB, RM, TLK, MMR. Data acquisition: EGG, TM, CBR, RMS. Formal analysis: EGG, CBR, TLK. Funding acquisition: SCM, TLK, J‐AJ. Investigation: EGG, TLK, MMR, SCM, CBR. Methodology: CBR, TLK, EGG. Supervision: TLK, SCM, MMR Visualization: EGG, CBR, CK. Writing—original draft preparation: EGG, TLK. Writing—review and editing: EGG, TLK, MMR, SCM, CBR, JSM, RMS, J‐AJ, CK, RM.

## Supporting information


**Figure S1a.** Allometric scaling of gorilla metacarpals (Log[metacarpal length] vs. Log[Body Mass]).

**Figure S1b.** Allometric scaling of gorilla proximal phalanges (Log[proximal phalangeal length] vs. Log[Body Mass]).

**Table S1.** Sensitivity of pairwise Wilcoxon tests of population differences in metacarpal and proximal phalangeal lengths to simulated inter‐observer measurement error.
**Table S2.** Sensitivity of ANCOVA interaction *p*‐values to simulated measurement error in Bwindi gorilla long bones.
**Table S4.** Results of pairwise population (intercept) comparisons from standardized major axis (SMA) regressions of log metacarpal length on log body mass.
**Table S5.** Results of pairwise population (intercept) comparisons from standardized major axis (SMA) regressions of log proximal hand phalanx length on log body mass.
**Table S6.** Pairwise comparisons of residual lengths for male metacarpals across gorilla populations (log [humeral + radial length]).
**Table S7.** Pairwise comparisons of residual lengths for female metacarpals across gorilla populations (log [humeral + radial length]).
**Table S8.** Pairwise comparisons of residual lengths for male proximal phalanges across gorilla populations (log [humeral + radial length]).
**Table S9.** Pairwise comparisons of residual lengths for female proximal phalanges across gorilla populations (log [humeral + radial length]).
**Table S10.** Kruskal–Wallis and pairwise Wilcoxon test results (Holm‐adjusted) comparing residuals from RMA regressions of log‐transformed proximal phalanx length on metacarpal length across gorilla populations.
**Table S11.** Mean and median residuals from RMA regressions of log‐transformed proximal phalanx length on metacarpal length (PP3~Mc3) by population and sex.

Data S1.

## Data Availability

The data that supports the findings of this study are available in the Supporting Information of this article.
