# Supplementary figures and images for: Frequency of arboreality is correlated with longer hand skeletons in Gorilla: Analysis of a new skeletal sample of Bwindi mountain gorillas
Source: J Anat. 2026 Apr 19;249(3):544–64. doi: 10.1111/joa.70121 (PMC13398981; doi:10.1111/joa.70121)

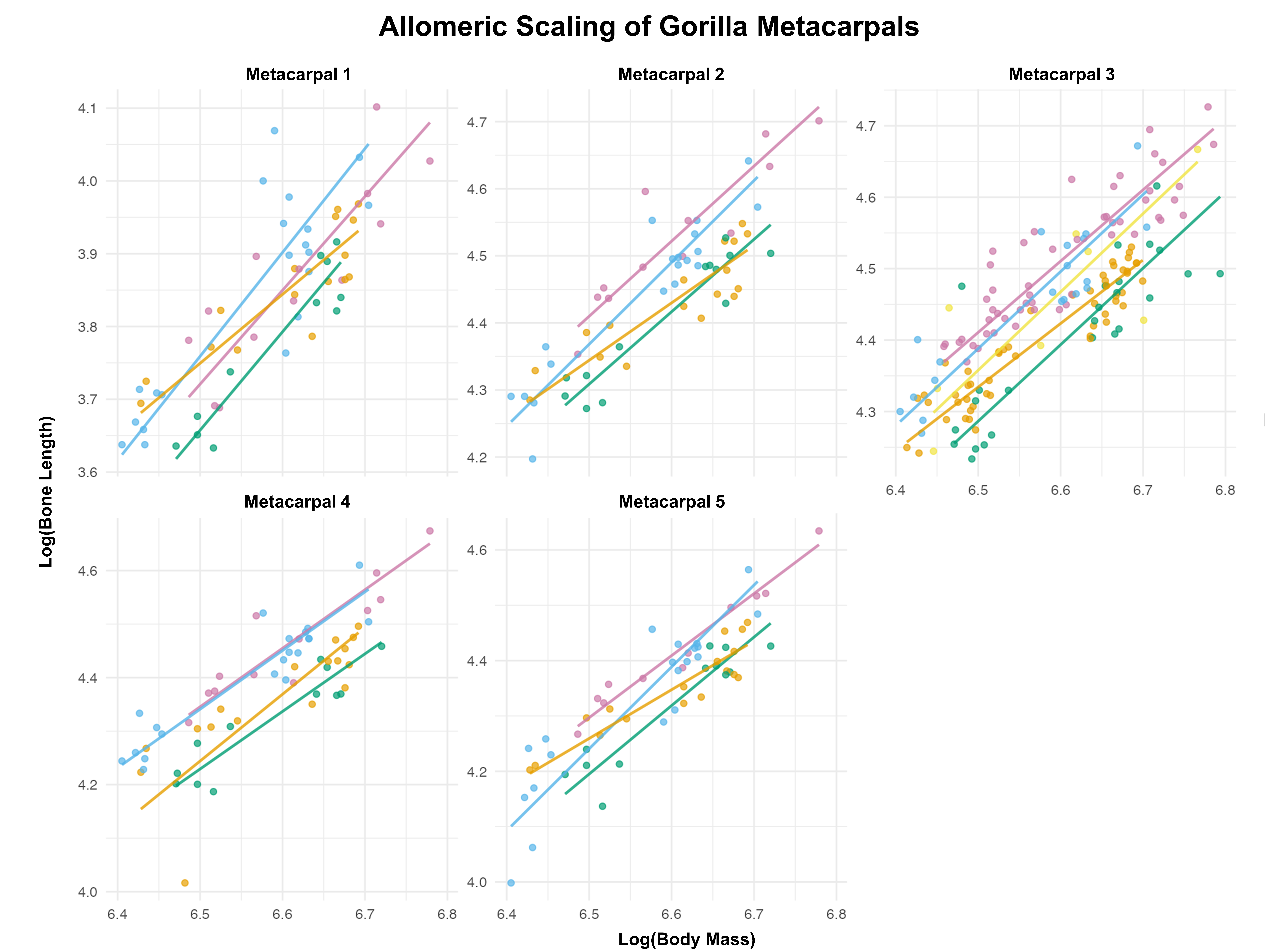

Supplement: Supplementary file 1 — Figure S1a. Allometric scaling of gorilla metacarpals (Log[metacarpal length] vs. Log[Body Mass]). [file JOA-249-544-s004.tif]

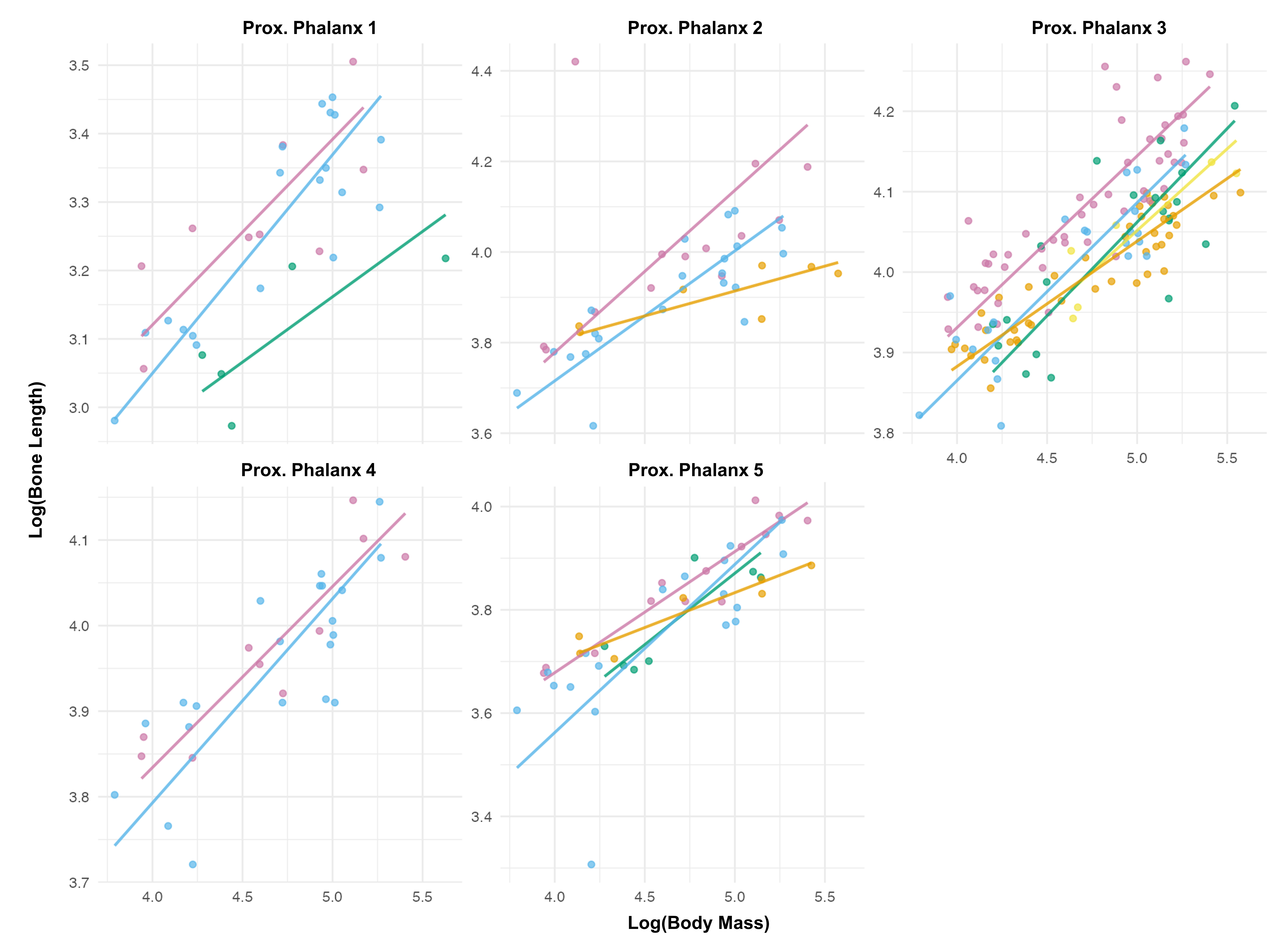

Supplement: Supplementary file 2 — Figure S1b. Allometric scaling of gorilla proximal phalanges (Log[proximal phalangeal length] vs. Log[Body Mass]). [file JOA-249-544-s003.tif]
