# Supplementary material for: Frequency of arboreality is correlated with longer hand skeletons in Gorilla: Analysis of a new skeletal sample of Bwindi mountain gorillas
Source: J Anat. 2026 Apr 19;249(3):544–64. doi: 10.1111/joa.70121 (PMC13398981; doi:10.1111/joa.70121)
Supplement: Supplementary file 3 — Table S1. Sensitivity of pairwise Wilcoxon tests of population differences in metacarpal and proximal phalangeal lengths to simulated inter‐observer measurement error. Table S2. Sensitivity of ANCOVA interaction p‐values to simulated measurement error in Bwindi gorilla long bones. Table S4. Results of pairwise population (intercept) comparisons from standardized major axis (SMA) regressions of log metacarpal length on log body mass. Table S5. Results of pairwise population (intercept) comparisons from standardized major axis (SMA) regressions of log proximal hand phalanx length on log body mass. Table S6. Pairwise comparisons of residual lengths for male metacarpals across gorilla populations (log [humeral + radial length]). Table S7. Pairwise comparisons of residual lengths for female metacarpals across gorilla populations (log [humeral + radial length]). Table S8. Pairwise comparisons of residual lengths for male proximal phalanges across gorilla populations (log [humeral + radial length]). Table S9. Pairwise comparisons of residual lengths for female proximal phalanges across gorilla populations (log [humeral + radial length]). Table S10. Kruskal–Wallis and pairwise Wilcoxon test results (Holm‐adjusted) comparing residuals from RMA regressions of log‐transformed proximal phalanx length on metacarpal length across gorilla populations. Table S11. Mean and median residuals from RMA regressions of log‐transformed proximal phalanx length on metacarpal length (PP3~Mc3) by population and sex. [file JOA-249-544-s001.docx]

**Supplement**

| **Bone** | **Perturbation** | **n tests** | **Min p** | **Median p** | **Max p** | **Fraction p < 0.05** |
| --- | --- | --- | --- | --- | --- | --- |
| PP1MAXLN | Additive (-1 mm) | 4 | 0.102 | 0.158 | 0.214 | <0.001 |
| PP1MAXLN | Additive (-2 mm) | 4 | 0.043 | 0.135 | 0.227 | 0.5 |
| PP1MAXLN | Additive (-3 mm) | 4 | 0.006 | 0.288 | 0.57 | 0.5 |
| PP1MAXLN | Additive (1 mm) | 4 | 0.023 | 0.511 | 1 | 0.5 |
| PP1MAXLN | Additive (2 mm) | 4 | 0.01 | 0.185 | 0.36 | 0.5 |
| PP1MAXLN | Additive (3 mm) | 4 | 0.001 | 0.064 | 0.126 | 0.5 |
| PP1MAXLN | Hybrid (+3 mm ± noise, SD=1 mm) | 4 | 0.003 | 0.095 | 0.187 | 0.5 |
| PP1MAXLN | Multiplicative (×0.97) | 4 | 0.065 | 0.166 | 0.267 | <0.001 |
| PP1MAXLN | Multiplicative (×0.99) | 4 | 0.039 | 0.225 | 0.41 | 0.5 |
| PP1MAXLN | Noise (SD=1mm) | 4 | 0.055 | 0.218 | 0.381 | <0.001 |
| PP1MAXLN | baseline | 4 | 0.028 | 0.258 | 0.488 | 0.5 |
| PP2MAXLN | Additive (-1 mm) | 4 | 0.003 | 0.473 | 0.942 | 0.5 |
| PP2MAXLN | Additive (-2 mm) | 4 | 0.001 | 0.288 | 0.575 | 0.5 |
| PP2MAXLN | Additive (-3 mm) | 4 | <0.001 | 0.197 | 0.393 | 0.5 |
| PP2MAXLN | Additive (1 mm) | 4 | 0.064 | 0.257 | 0.449 | <0.001 |
| PP2MAXLN | Additive (2 mm) | 4 | 0.152 | 0.182 | 0.213 | <0.001 |
| PP2MAXLN | Additive (3 mm) | 4 | 0.102 | 0.221 | 0.339 | <0.001 |
| PP2MAXLN | Hybrid (+3 mm ± noise, SD=1 mm) | 4 | 0.067 | 0.203 | 0.339 | <0.001 |
| PP2MAXLN | Multiplicative (×0.97) | 4 | 0.002 | 0.376 | 0.751 | 0.5 |
| PP2MAXLN | Multiplicative (×0.99) | 4 | 0.007 | 0.455 | 0.903 | 0.5 |
| PP2MAXLN | Noise (SD=1mm) | 4 | 0.009 | 0.418 | 0.826 | 0.5 |
| PP2MAXLN | baseline | 4 | 0.018 | 0.403 | 0.788 | 0.5 |
| PP3MAXLN | Additive (-1 mm) | 4 | 0.001 | 0.146 | 0.788 | 0.25 |
| PP3MAXLN | Additive (-2 mm) | 4 | <0.001 | 0.068 | 0.306 | 0.25 |
| PP3MAXLN | Additive (-3 mm) | 4 | <0.001 | 0.03 | 0.089 | 0.75 |
| PP3MAXLN | Additive (1 mm) | 4 | 0.048 | 0.437 | 0.874 | 0.25 |
| PP3MAXLN | Additive (2 mm) | 4 | 0.039 | 0.456 | 0.844 | 0.25 |
| PP3MAXLN | Additive (3 mm) | 4 | 0.004 | 0.369 | 0.806 | 0.25 |
| PP3MAXLN | Hybrid (+3 mm ± noise, SD=1 mm) | 4 | 0.006 | 0.391 | 0.922 | 0.25 |
| PP3MAXLN | Multiplicative (×0.97) | 4 | <0.001 | 0.091 | 0.443 | 0.25 |
| PP3MAXLN | Multiplicative (×0.99) | 4 | 0.003 | 0.212 | 0.968 | 0.25 |
| PP3MAXLN | Noise (SD=1mm) | 4 | 0.007 | 0.364 | 0.619 | 0.25 |
| PP3MAXLN | baseline | 4 | 0.008 | 0.33 | 0.667 | 0.25 |
| PP4MAXLN | Additive (-1 mm) | 4 | 0.155 | 0.155 | 0.155 | <0.001 |
| PP4MAXLN | Additive (-2 mm) | 4 | 0.045 | 0.045 | 0.045 | 1 |
| PP4MAXLN | Additive (-3 mm) | 4 | 0.01 | 0.01 | 0.01 | 1 |
| PP4MAXLN | Additive (1 mm) | 4 | 0.799 | 0.799 | 0.799 | <0.001 |
| PP4MAXLN | Additive (2 mm) | 4 | 0.846 | 0.846 | 0.846 | <0.001 |
| PP4MAXLN | Additive (3 mm) | 4 | 0.402 | 0.402 | 0.402 | <0.001 |
| PP4MAXLN | Hybrid (+3 mm ± noise, SD=1 mm) | 4 | 0.313 | 0.313 | 0.313 | <0.001 |
| PP4MAXLN | Multiplicative (×0.97) | 4 | 0.091 | 0.091 | 0.091 | <0.001 |
| PP4MAXLN | Multiplicative (×0.99) | 4 | 0.269 | 0.269 | 0.269 | <0.001 |
| PP4MAXLN | Noise (SD=1mm) | 4 | 0.402 | 0.402 | 0.402 | <0.001 |
| PP4MAXLN | baseline | 4 | 0.375 | 0.375 | 0.375 | <0.001 |
| PP5MAXLN | Additive (-1 mm) | 4 | 0.007 | 0.389 | 0.486 | 0.33 |
| PP5MAXLN | Additive (-2 mm) | 4 | 0.002 | 0.232 | 0.29 | 0.33 |
| PP5MAXLN | Additive (-3 mm) | 4 | <0.001 | 0.127 | 0.173 | 0.33 |
| PP5MAXLN | Additive (1 mm) | 4 | 0.075 | 0.976 | 1 | <0.001 |
| PP5MAXLN | Additive (2 mm) | 4 | 0.21 | 0.486 | 0.739 | <0.001 |
| PP5MAXLN | Additive (3 mm) | 4 | 0.255 | 0.348 | 0.575 | <0.001 |
| PP5MAXLN | Hybrid (+3 mm ± noise, SD=1 mm) | 4 | 0.348 | 0.359 | 0.625 | <0.001 |
| PP5MAXLN | Multiplicative (×0.97) | 4 | 0.003 | 0.331 | 0.414 | 0.33 |
| PP5MAXLN | Multiplicative (×0.99) | 4 | 0.015 | 0.523 | 0.565 | 0.33 |
| PP5MAXLN | Noise (SD=1mm) | 4 | 0.039 | 0.785 | 0.89 | 0.33 |
| PP5MAXLN | baseline | 4 | 0.025 | 0.694 | 0.718 | 0.33 |
| Mc1MAXLN | Additive (-1 mm) | 4 | 0.15 | 0.513 | 0.965 | <0.001 |
| Mc1MAXLN | Additive (-2 mm) | 4 | 0.055 | 0.536 | 0.827 | <0.001 |
| Mc1MAXLN | Additive (-3 mm) | 4 | 0.022 | 0.247 | 0.627 | 0.33 |
| Mc1MAXLN | Additive (1 mm) | 4 | 0.087 | 0.101 | 0.84 | <0.001 |
| Mc1MAXLN | Additive (2 mm) | 4 | 0.025 | 0.036 | 0.748 | 0.67 |
| Mc1MAXLN | Additive (3 mm) | 4 | 0.003 | 0.023 | 0.373 | 0.67 |
| McC1MAXLN | Hybrid (+3 mm ± noise, SD=1 mm) | 4 | 0.007 | 0.03 | 0.393 | 0.67 |
| Mc1MAXLN | Multiplicative (×0.97) | 4 | 0.1 | 0.603 | 0.838 | <0.001 |
| Mc1MAXLN | Multiplicative (×0.99) | 4 | 0.3 | 0.356 | 0.66 | <0.001 |
| Mc1MAXLN | Noise (SD=1mm) | 4 | 0.144 | 0.279 | 0.614 | <0.001 |
| Mc1MAXLN | baseline | 4 | 0.208 | 0.414 | 0.491 | <0.001 |
| Mc2MAXLN | Additive (-1 mm) | 4 | 0.007 | 0.291 | 0.449 | 0.33 |
| Mc2MAXLN | Additive (-2 mm) | 4 | 0.004 | 0.565 | 0.792 | 0.33 |
| Mc2MAXLN | Additive (-3 mm) | 4 | 0.002 | 0.955 | 1 | 0.33 |
| Mc2MAXLN | Additive (1 mm) | 4 | 0.029 | 0.061 | 0.087 | 0.33 |
| Mc2MAXLN | Additive (2 mm) | 4 | 0.02 | 0.043 | 0.048 | 1 |
| Mc2MAXLN | Additive (3 mm) | 4 | 0.005 | 0.019 | 0.067 | 0.67 |
| Mc2MAXLN | Hybrid (+3 mm ± noise, SD=1 mm) | 4 | 0.005 | 0.019 | 0.056 | 0.67 |
| Mc2MAXLN | Multiplicative (×0.97) | 4 | 0.002 | 0.783 | 0.947 | 0.33 |
| Mc2MAXLN | Multiplicative (×0.99) | 4 | 0.008 | 0.277 | 0.429 | 0.33 |
| Mc2MAXLN | Noise (SD=1mm) | 4 | 0.014 | 0.133 | 0.177 | 0.33 |
| Mc2MAXLN | baseline | 4 | 0.018 | 0.143 | 0.193 | 0.33 |
| Mc3MAXLN | Additive (-1 mm) | 4 | 0.009 | 0.378 | 0.668 | 0.25 |
| Mc3MAXLN | Additive (-2 mm) | 4 | 0.002 | 0.47 | 0.819 | 0.25 |
| Mc3MAXLN | Additive (-3 mm) | 4 | <0.001 | 0.566 | 0.898 | 0.25 |
| Mc3MAXLN | Additive (1 mm) | 4 | 0.035 | 0.1 | 0.953 | 0.25 |
| Mc3MAXLN | Additive (2 mm) | 4 | 0.01 | 0.111 | 0.785 | 0.25 |
| Mc3MAXLN | Additive (3 mm) | 4 | 0.002 | 0.15 | 0.682 | 0.5 |
| Mc3MAXLN | Hybrid (+3 mm ± noise, SD=1 mm) | 4 | 0.002 | 0.136 | 0.612 | 0.5 |
| Mc3MAXLN | Multiplicative (×0.97) | 4 | <0.001 | 0.489 | 1 | 0.25 |
| Mc3MAXLN | Multiplicative (×0.99) | 4 | 0.009 | 0.347 | 0.725 | 0.25 |
| Mc3MAXLN | Noise (SD=1mm) | 4 | 0.026 | 0.209 | 1 | 0.25 |
| Mc3MAXLN | baseline | 4 | 0.027 | 0.206 | 0.953 | 0.25 |
| Mc4MAXLN | Additive (-1 mm) | 4 | 0.028 | 0.049 | 0.071 | 0.67 |
| Mc4MAXLN | Additive (-2 mm) | 4 | 0.026 | 0.073 | 0.239 | 0.33 |
| Mc4MAXLN | Additive (-3 mm) | 4 | 0.014 | 0.179 | 0.496 | 0.33 |
| Mc4MAXLN | Additive (1 mm) | 4 | 0.007 | 0.016 | 0.153 | 0.67 |
| Mc4MAXLN | Additive (2 mm) | 4 | 0.001 | 0.009 | 0.24 | 0.67 |
| Mc4MAXLN | Additive (3 mm) | 4 | <0.001 | 0.005 | 0.398 | 0.67 |
| Mc4MAXLN | Hybrid (+3 mm ± noise, SD=1 mm) | 4 | <0.001 | 0.005 | 0.367 | 0.67 |
| Mc4MAXLN | Multiplicative (×0.97) | 4 | 0.021 | 0.127 | 0.324 | 0.33 |
| Mc4MAXLN | Multiplicative (×0.99) | 4 | 0.028 | 0.051 | 0.059 | 0.33 |
| Mc4MAXLN | Noise (SD=1mm) | 4 | 0.02 | 0.026 | 0.086 | 0.67 |
| Mc4MAXLN | baseline | 4 | 0.023 | 0.024 | 0.087 | 0.67 |
| Mc5MAXLN | Additive (-1 mm) | 4 | 0.017 | 0.668 | 0.893 | 0.33 |
| Mc5MAXLN | Additive (-2 mm) | 4 | 0.008 | 0.827 | 0.895 | 0.33 |
| Mc5MAXLN | Additive (-3 mm) | 4 | 0.005 | 0.529 | 0.557 | 0.33 |
| Mc5MAXLN | Additive (1 mm) | 4 | 0.041 | 0.188 | 0.247 | 0.33 |
| Mc5MAXLN | Additive (2 mm) | 4 | 0.071 | 0.089 | 0.185 | <0.001 |
| Mc5MAXLN | Additive (3 mm) | 4 | 0.047 | 0.097 | 0.1 | 0.33 |
| Mc5MAXLN | Hybrid (+3 mm ± noise, SD=1 mm) | 4 | 0.056 | 0.097 | 0.097 | <0.001 |
| Mc5MAXLN | Multiplicative (×0.97) | 4 | 0.006 | 0.72 | 0.75 | 0.33 |
| Mc5MAXLN | Multiplicative (×0.99) | 4 | 0.019 | 0.566 | 0.827 | 0.33 |
| MC5MAXLN | Noise (SD=1mm) | 4 | 0.025 | 0.438 | 0.651 | 0.33 |
| MC5MAXLN | baseline | 4 | 0.026 | 0.374 | 0.411 | 0.33 |

**Table S1**. **Sensitivity of pairwise Wilcoxon tests of population differences in metacarpal and proximal phalangeal lengths to simulated inter-observer measurement error.** Each row shows results for one element under a given perturbation scenario. Perturbations included fixed additive offsets (±1–3 mm), multiplicative scalars (0.97, 0.99), Gaussian noise (SD = 1 mm), and hybrid offsets (+3 mm ± noise). For each bone and perturbation, we report the number of contrasts tested (n), the minimum, median, and maximum p-values across contrasts, and the fraction of contrasts with p < 0.05. Baseline rows show unperturbed data. MAXLN=Maximum Length.

| **Pair** | **Perturbation** | **n tests** | **Min p** | **Median p** | **Max p** | **Fraction p < 0.05** |
| --- | --- | --- | --- | --- | --- | --- |
| RH | Additive (-1 mm) | 1 | 0.006 | 0.006 | 0.006 | 1 |
| RH | Additive (-3 mm) | 1 | 0.006 | 0.006 | 0.006 | 1 |
| RH | Additive (-5 mm) | 1 | 0.006 | 0.006 | 0.006 | 1 |
| RH | Additive (1 mm) | 1 | 0.007 | 0.007 | 0.007 | 1 |
| RH | Additive (10 mm) | 1 | 0.008 | 0.008 | 0.008 | 1 |
| RH | Additive (3 mm) | 1 | 0.007 | 0.007 | 0.007 | 1 |
| RH | Additive (5 mm) | 1 | 0.007 | 0.007 | 0.007 | 1 |
| RH | Hybrid (+10 mm ± noise, SD=2 mm) | 1000 | 0.007 | 0.007 | 0.007 | 1 |
| RH | Multiplicative (×0.9) | 1 | 0.007 | 0.007 | 0.007 | 1 |
| RH | Multiplicative (×0.95) | 1 | 0.007 | 0.007 | 0.007 | 1 |
| RH | Multiplicative (×0.97) | 1 | 0.007 | 0.007 | 0.007 | 1 |
| RH | Multiplicative (×0.99) | 1 | 0.007 | 0.007 | 0.007 | 1 |
| RH | Split offsets (fore=-5 mm; hind=-5 mm) | 1 | 0.006 | 0.006 | 0.006 | 1 |
| RH | Split offsets (fore=-5 mm; hind=0 mm) | 1 | 0.006 | 0.006 | 0.006 | 1 |
| RH | Split offsets (fore=-5 mm; hind=10 mm) | 1 | 0.006 | 0.006 | 0.006 | 1 |
| RH | Split offsets (fore=-5 mm; hind=5 mm) | 1 | 0.006 | 0.006 | 0.006 | 1 |
| RH | Split offsets (fore=0 mm; hind=-5 mm) | 1 | 0.007 | 0.007 | 0.007 | 1 |
| RH | Split offsets (fore=0 mm; hind=0 mm) | 1 | 0.007 | 0.007 | 0.007 | 1 |
| RH | Split offsets (fore=0 mm; hind=10 mm) | 1 | 0.007 | 0.007 | 0.007 | 1 |
| RH | Split offsets (fore=0 mm; hind=5 mm) | 1 | 0.007 | 0.007 | 0.007 | 1 |
| RH | Split offsets (fore=10 mm; hind=-5 mm) | 1 | 0.008 | 0.008 | 0.008 | 1 |
| RH | Split offsets (fore=10 mm; hind=0 mm) | 1 | 0.008 | 0.008 | 0.008 | 1 |
| RH | Split offsets (fore=10 mm; hind=10 mm) | 1 | 0.008 | 0.008 | 0.008 | 1 |
| RH | Split offsets (fore=10 mm; hind=5 mm) | 1 | 0.008 | 0.008 | 0.008 | 1 |
| RH | Split offsets (fore=5 mm; hind=-5 mm) | 1 | 0.007 | 0.007 | 0.007 | 1 |
| RH | Split offsets (fore=5 mm; hind=0 mm) | 1 | 0.007 | 0.007 | 0.007 | 1 |
| RH | Split offsets (fore=5 mm; hind=10 mm) | 1 | 0.007 | 0.007 | 0.007 | 1 |
| RH | Split offsets (fore=5 mm; hind=5 mm) | 1 | 0.007 | 0.007 | 0.007 | 1 |
| TF | Additive (-1 mm) | 1 | <0.001 | <0.001 | <0.001 | 1 |
| TF | Additive (-3 mm) | 1 | 0.001 | 0.001 | 0.001 | 1 |
| TF | Additive (-5 mm) | 1 | 0.001 | 0.001 | 0.001 | 1 |
| TF | Additive (1 mm) | 1 | <0.001 | <0.001 | <0.001 | 1 |
| TF | Additive (10 mm) | 1 | <0.001 | <0.001 | <0.001 | 1 |
| TF | Additive (3 mm) | 1 | <0.001 | <0.001 | <0.001 | 1 |
| TF | Additive (5 mm) | 1 | <0.001 | <0.001 | <0.001 | 1 |
| TF | Hybrid (+10 mm ± noise, SD=2 mm) | 1000 | <0.001 | <0.001 | <0.001 | 1 |
| TF | Multiplicative (×0.9) | 1 | <0.001 | <0.001 | <0.001 | 1 |
| TF | Multiplicative (×0.95) | 1 | <0.001 | <0.001 | <0.001 | 1 |
| TF | Multiplicative (×0.97) | 1 | <0.001 | <0.001 | <0.001 | 1 |
| TF | Multiplicative (×0.99) | 1 | <0.001 | <0.001 | <0.001 | 1 |
| TF | Split offsets (fore=-5 mm; hind=-5 mm) | 1 | 0.001 | 0.001 | 0.001 | 1 |
| TF | Split offsets (fore=-5 mm; hind=0 mm) | 1 | <0.001 | <0.001 | <0.001 | 1 |
| TF | Split offsets (fore=-5 mm; hind=10 mm) | 1 | <0.001 | <0.001 | <0.001 | 1 |
| TF | Split offsets (fore=-5 mm; hind=5 mm) | 1 | <0.001 | <0.001 | <0.001 | 1 |
| TF | Split offsets (fore=0 mm; hind=-5 mm) | 1 | 0.001 | 0.001 | 0.001 | 1 |
| TF | Split offsets (fore=0 mm; hind=0 mm) | 1 | <0.001 | <0.001 | <0.001 | 1 |
| TF | Split offsets (fore=0 mm; hind=10 mm) | 1 | <0.001 | <0.001 | <0.001 | 1 |
| TF | Split offsets (fore=0 mm; hind=5 mm) | 1 | <0.001 | <0.001 | <0.001 | 1 |
| TF | Split offsets (fore=10 mm; hind=-5 mm) | 1 | 0.001 | 0.001 | 0.001 | 1 |
| TF | Split offsets (fore=10 mm; hind=0 mm) | 1 | <0.001 | <0.001 | <0.001 | 1 |
| TF | Split offsets (fore=10 mm; hind=10 mm) | 1 | <0.001 | <0.001 | <0.001 | 1 |
| TF | Split offsets (fore=10 mm; hind=5 mm) | 1 | <0.001 | <0.001 | <0.001 | 1 |
| TF | Split offsets (fore=5 mm; hind=-5 mm) | 1 | 0.001 | 0.001 | 0.001 | 1 |
| TF | Split offsets (fore=5 mm; hind=0 mm) | 1 | <0.001 | <0.001 | <0.001 | 1 |
| TF | Split offsets (fore=5 mm; hind=10 mm) | 1 | <0.001 | <0.001 | <0.001 | 1 |
| TF | Split offsets (fore=5 mm; hind=5 mm) | 1 | <0.001 | <0.001 | <0.001 | 1 |

**Table S2. Sensitivity of ANCOVA interaction p-values to simulated measurement error in Bwindi gorilla long bones.** Maximum humerus, radius, femur, and tibia lengths were re-analyzed after applying artificial perturbations only to Bwindi specimens. Perturbations included fixed additive shifts (±1–10 mm), multiplicative scalars (0.90–0.99), hybrid offsets (+10 mm plus Gaussian noise, SD = 2 mm), and split offsets (different adjustments for forelimb vs. hindlimb). For each perturbed dataset, the log–log ANCOVA model tested whether Bwindi scaling differed from other groups. RH=Radius and humerus; TF= Tibia and Femur.

| **Bone** | **Population 1** | **Population 2** | **n (Gr. 1)** | **n (Gr. 2)** | **p value** | **Significance Level** |
| --- | --- | --- | --- | --- | --- | --- |
| Ray 2 | Bwindi | Virunga | 13 | 6 | 0.0786 |  |
| Ray 2 | Lowland | Bwindi | 11 | 13 | 0.0929 |  |
| Ray 2 | Lowland | Virunga | 11 | 6 | 0.0414 | * |
| Ray 3 | Bwindi | Virunga | 17 | 11 | 0.0253 | * |
| Ray 3 | Lowland | Bwindi | 12 | 17 | 0.150 |  |
| Ray 3 | Lowland | Virunga | 12 | 11 | 0.00191 | ** |
| Ray 4 | Lowland | Bwindi | 9 | 13 | 0.689 |  |
| Ray 5 | Bwindi | Virunga | 10 | 7 | 0.814 |  |
| Ray 5 | Lowland | Bwindi | 10 | 10 | 0.814 |  |
| Ray 5 | Lowland | Virunga | 10 | 7 | 0.471 |  |

**Table S3.** Pairwise Wilcoxon rank-sum comparisons of size-adjusted total (MC+PP+IP) lengths across gorilla elevation groups, separated by ray. Values shown are raw and Holm–Bonferroni–adjusted p-values; only group pairs with ≥5 individuals in each group are reported.

| **Bone** | **Population 1** | **Population 2** | **Test Statistic** | **df** | **p_adj_BH** | **Significance Level** |
| --- | --- | --- | --- | --- | --- | --- |
| McI | Bwindi | High | 9.135 | 1 | 0.0108 | * |
| McI | Bwindi | Low | 10.579 | 1 | 0.0091 | ** |
| McI | Bwindi | Lowland | 0.013 | 1 | 0.9092 |  |
| McI | Bwindi | Virunga | 2.430 | 1 | 0.1733 |  |
| McI | High | Low | 2.046 | 1 | 0.1733 |  |
| McI | High | Lowland | 8.158 | 1 | 0.0132 | * |
| McI | High | Virunga | 4.310 | 1 | 0.0677 |  |
| McI | Low | Lowland | 10.297 | 1 | 0.0091 | ** |
| McI | Low | Virunga | 7.180 | 1 | 0.0174 | * |
| McI | Lowland | Virunga | 2.097 | 1 | 0.1733 |  |
| Mc2 | Bwindi | High | 4.899 | 1 | 0.0440 | * |
| Mc2 | Bwindi | Lowland | 9.241 | 1 | 0.0061 | ** |
| Mc2 | Bwindi | Virunga | 4.291 | 1 | 0.0493 | * |
| Mc2 | High | Lowland | 21.596 | 1 | 0.0000 | *** |
| Mc2 | High | Virunga | 0.579 | 1 | 0.4487 |  |
| Mc2 | Lowland | Virunga | 25.424 | 1 | 0.0000 | *** |
| Mc3 | Bwindi | High | 16.960 | 1 | 0.0002 | *** |
| Mc3 | Bwindi | Low | 6.350 | 1 | 0.0212 | * |
| Mc3 | Bwindi | Lowland | 6.020 | 1 | 0.0217 | * |
| Mc3 | Bwindi | Virunga | 8.812 | 1 | 0.0069 | ** |
| Mc3 | High | Low | 0.196 | 1 | 0.6586 |  |
| Mc3 | High | Lowland | 55.347 | 1 | 0.0000 | *** |
| Mc3 | High | Virunga | 3.335 | 1 | 0.0871 |  |
| Mc3 | Low | Lowland | 19.416 | 1 | 0.0001 | *** |
| Mc3 | Low | Virunga | 0.518 | 1 | 0.5254 |  |
| Mc3 | Lowland | Virunga | 47.737 | 1 | 0.0000 | *** |
| Mc4 | Bwindi | High | 7.970 | 1 | 0.0145 | * |
| Mc4 | Bwindi | Low | 7.130 | 1 | 0.0149 | * |
| Mc4 | Bwindi | Lowland | 1.918 | 1 | 0.2117 |  |
| Mc4 | Bwindi | Virunga | 7.300 | 1 | 0.0149 | * |
| Mc4 | High | Low | 0.974 | 1 | 0.3344 |  |
| Mc4 | High | Lowland | 13.848 | 1 | 0.0017 | ** |
| Mc4 | High | Virunga | 0.941 | 1 | 0.3344 |  |
| Mc4 | Low | Lowland | 10.905 | 1 | 0.0045 | ** |
| Mc4 | Low | Virunga | 2.550 | 1 | 0.1624 |  |
| Mc4 | Lowland | Virunga | 14.090 | 1 | 0.0017 | ** |
| Mc5 | Bwindi | High | 2.198 | 1 | 0.2023 |  |
| Mc5 | Bwindi | Low | 4.443 | 1 | 0.0756 |  |
| Mc5 | Bwindi | Lowland | 7.816 | 1 | 0.0158 | * |
| Mc5 | Bwindi | Virunga | 0.319 | 1 | 0.5736 |  |
| Mc5 | High | Low | 1.410 | 1 | 0.2746 |  |
| Mc5 | High | Lowland | 13.856 | 1 | 0.0024 | ** |
| Mc5 | High | Virunga | 1.357 | 1 | 0.2746 |  |
| Mc5 | Low | Lowland | 12.297 | 1 | 0.0024 | ** |
| Mc5 | Low | Virunga | 3.689 | 1 | 0.0965 |  |
| Mc5 | Lowland | Virunga | 12.412 | 1 | 0.0024 | ** |

**Table S4**. Results of pairwise population (intercept) comparisons from standardized major axis (SMA) regressions of log metacarpal length on log body mass. Each row shows the test statistic, degrees of freedom, and Benjamini–Hochberg adjusted p-value for a comparison between two gorilla populations. Analyses were performed using SMA models with a common slope fitted to log₁₀-transformed and mean-centered body mass. Significant adjusted p-values (p < 0.05) indicate that populations differ in regression line elevation (intercept), i.e., relative metacarpal length at a given body mass, under the assumption of a common slope.

| **Bone** | **Population 1** | | **Population 2** | | **test_stat** | | **df** | **p_value_BH** | **Significance Level** |
| --- | --- | --- | --- | --- | --- | --- | --- | --- | --- |
|  |  |  | |  | |  | |  |  |
| PPI | Bwindi | | High | | 6.372 | | 1 | 0.0246 | * |
| PPI | Bwindi | | Lowland | | 0.562 | | 1 | 0.4584 |  |
| PPI | High | | Lowland | | 7.945 | | 1 | 0.0239 | * |
| PP2 | Bwindi | | High | | 0.773 | | 1 | 0.4612 |  |
| PP2 | Bwindi | | Lowland | | 4.685 | | 1 | 0.0957 |  |
| PP2 | Bwindi | | Virunga | | 1.004 | | 1 | 0.4612 |  |
| PP2 | High | | Lowland | | 4.162 | | 1 | 0.0957 |  |
| PP2 | High | | Virunga | | 0.023 | | 1 | 0.8793 |  |
| PP2 | Lowland | | Virunga | | 6.565 | | 1 | 0.0850 |  |
| PP3 | Bwindi | | High | | 0.840 | | 1 | 0.6017 |  |
| PP3 | Bwindi | | Low | | 0.649 | | 1 | 0.6026 |  |
| PP3 | Bwindi | | Lowland | | 13.501 | | 1 | 0.0011 | ** |
| PP3 | Bwindi | | Virunga | | 2.353 | | 1 | 0.2548 |  |
| PP3 | High | | Low | | 0.024 | | 1 | 0.9025 |  |
| PP3 | High | | Lowland | | 20.758 | | 1 | 0.0001 | *** |
| PP3 | High | | Virunga | | 0.188 | | 1 | 0.8321 |  |
| PP3 | Low | | Lowland | | 9.980 | | 1 | 0.0049 | ** |
| PP3 | Low | | Virunga | | 0.015 | | 1 | 0.9025 |  |
| PP3 | Lowland | | Virunga | | 39.800 | | 1 | 0.0000 | *** |
| PP4 | Bwindi | | High | | 0.649 | | 1 | 0.4263 |  |
| PP4 | Bwindi | | Lowland | | 0.975 | | 1 | 0.4263 |  |
| PP4 | High | | Lowland | | 1.773 | | 1 | 0.4263 |  |
| PP5 | Bwindi | | High | | 0.029 | | 1 | 0.9362 |  |
| PP5 | Bwindi | | Lowland | | 3.620 | | 1 | 0.3828 |  |
| PP5 | Bwindi | | Virunga | | 0.071 | | 1 | 0.9362 |  |
| PP5 | High | | Lowland | | 1.733 | | 1 | 0.4549 |  |
| PP5 | High | | Virunga | | 0.006 | | 1 | 0.9362 |  |
| PP5 | Lowland | | Virunga | | 1.499 | | 1 | 0.4549 |  |

**Table S5**. Results of pairwise population (intercept) comparisons from standardized major axis (SMA) regressions of log proximal hand phalanx length on log body mass. Each row shows the test statistic, degrees of freedom, and Benjamini–Hochberg adjusted p-value for a comparison between two gorilla populations. Significant adjusted p-values (p < 0.05) indicate that the groups differ in regression line elevation (intercept), i.e., relative proximal phalanx length at a given body mass, under the assumption of a common slope.

| **Bone** | **Population 1** | **Population 2** | **Kruskal_p** | **Holm_adjusted_p** | **Significance level** |
| --- | --- | --- | --- | --- | --- |
|  |  |  |  |  |  |
| Mc2 | Virunga | Western Lowland | <0.001 | <0.001 | *** |
| Mc2 | Bwindi | High Grauer's | <0.001 | <0.001 | *** |
| Mc2 | Virunga | Bwindi | <0.001 | <0.001 | *** |
| Mc3 | High Grauer's | Western Lowland | <0.001 | <0.001 | *** |
| Mc3 | Virunga | Western Lowland | <0.001 | <0.001 | *** |
| Mc3 | Bwindi | High Grauer's | <0.001 | <0.001 | *** |
| Mc3 | Virunga | Bwindi | <0.001 | <0.001 | *** |
| Mc4 | Virunga | Western Lowland | <0.001 | <0.001 | *** |
| Mc4 | Virunga | Bwindi | <0.001 | <0.001 | *** |
| Mc5 | Virunga | Western Lowland | <0.001 | <0.001 | *** |
| Mc5 | Virunga | Bwindi | <0.001 | <0.001 | *** |
| Mc2 | High Grauer's | Western Lowland | <0.001 | 0.002 | ** |
| Mc4 | High Grauer's | Western Lowland | <0.001 | 0.004 | ** |
| Mc4 | Bwindi | High Grauer's | <0.001 | 0.003 | ** |
| Mc5 | High Grauer's | Western Lowland | <0.001 | 0.005 | ** |
| Mc1 | Bwindi | High Grauer's | 0.012 | 0.046 | * |
| Mc1 | Virunga | Bwindi | 0.012 | 0.013 | * |
| Mc5 | Bwindi | High Grauer's | <0.001 | 0.01 | * |
| Mc1 | High Grauer's | Western Lowland | 0.012 | 0.816 |  |
| Mc1 | Bwindi | Western Lowland | 0.012 | 0.506 |  |
| Mc1 | Virunga | Western Lowland | 0.012 | 0.952 |  |
| Mc1 | Virunga | High Grauer's | 0.012 | 0.952 |  |
| Mc2 | Bwindi | Western Lowland | <0.001 | 0.151 |  |
| Mc2 | Virunga | High Grauer's | <0.001 | 0.774 |  |
| Mc3 | Low Grauer's | Western Lowland | <0.001 | 0.705 |  |
| Mc3 | Bwindi | Western Lowland | <0.001 | 1 |  |
| Mc3 | High Grauer's | Low Grauer's | <0.001 | 0.528 |  |
| Mc3 | Bwindi | Low Grauer's | <0.001 | 1 |  |
| Mc3 | Virunga | Low Grauer's | <0.001 | 0.528 |  |
| Mc3 | Virunga | High Grauer's | <0.001 | 0.528 |  |
| Mc4 | Bwindi | Western Lowland | <0.001 | 0.69 |  |
| Mc4 | Virunga | High Grauer's | <0.001 | 0.195 |  |
| Mc5 | Bwindi | Western Lowland | <0.001 | 0.483 |  |
| Mc5 | Virunga | High Grauer's | <0.001 | 1 |  |

**Table S6.** Pairwise comparisons of residual lengths for male metacarpals across gorilla populations (log [humeral+radial length]). P-values are derived from Wilcoxon rank-sum tests with Holm correction for multiple comparisons, with significance determined at α=0.05. Only groups with ≥5 specimens per bone × elevation combination were included in analyses. Significant differences (p < 0.05) are indicated in the "Significance" column. 'High' refers to high elevation Grauer's gorillas, 'Low' to low elevation Grauer's gorillas, and 'lowland' to western lowland gorillas.

| **Bone** | **Population 1** | | **Population 2** | | **Kruskal_p** | | **Holm_adjusted_p** | | **Significance level** | | |
| --- | --- | --- | --- | --- | --- | --- | --- | --- | --- | --- | --- |
|  |  |  | |  | |  | |  | |  |  |
| Mc3 | High Grauer's | | Western Lowland | | <0.001 | | <0.001 | | *** | | |
| Mc3 | Virunga | | Western Lowland | | <0.001 | | <0.001 | | *** | | |
| Mc1 | Virunga | | High Grauer's | | 0.018 | | 0.002 | | ** | | |
| Mc2 | Virunga | | Western Lowland | | 0.001 | | 0.009 | | ** | | |
| Mc3 | Bwindi | | High Grauer's | | <0.001 | | 0.009 | | ** | | |
| Mc3 | Virunga | | High Grauer's | | <0.001 | | 0.005 | | ** | | |
| Mc3 | Virunga | | Bwindi | | <0.001 | | 0.004 | | ** | | |
| Mc4 | High Grauer's | | Western Lowland | | <0.001 | | 0.006 | | ** | | |
| Mc4 | Virunga | | Western Lowland | | <0.001 | | 0.002 | | ** | | |
| Mc4 | Bwindi | | High Grauer's | | <0.001 | | 0.005 | | ** | | |
| Mc4 | Virunga | | High Grauer's | | <0.001 | | 0.008 | | ** | | |
| Mc4 | Virunga | | Bwindi | | <0.001 | | 0.002 | | ** | | |
| Mc5 | Virunga | | Western Lowland | | 0.001 | | 0.007 | | ** | | |
| Mc1 | Bwindi | | High Grauer's | | 0.018 | | 0.013 | | * | | |
| Mc2 | High Grauer's | | Western Lowland | | 0.001 | | 0.022 | | * | | |
| Mc2 | Virunga | | High Grauer's | | 0.001 | | 0.026 | | * | | |
| Mc5 | High Grauer's | | Western Lowland | | 0.001 | | 0.013 | | * | | |
| Mc5 | Virunga | | High Grauer's | | 0.001 | | 0.013 | | * | | |
| Mc1 | High Grauer's | | Western Lowland | | 0.018 | | 0.502 | |  | | |
| Mc1 | Bwindi | | Western Lowland | | 0.018 | | 1 | |  | | |
| Mc1 | Virunga | | Western Lowland | | 0.018 | | 1 | |  | | |
| Mc1 | Virunga | | Bwindi | | 0.018 | | 1 | |  | | |
| Mc2 | Bwindi | | Western Lowland | | 0.001 | | 0.251 | |  | | |
| Mc2 | Bwindi | | High Grauer's | | 0.001 | | 0.123 | |  | | |
| Mc2 | Virunga | | Bwindi | | 0.001 | | 0.365 | |  | | |
| Mc3 | Bwindi | | Western Lowland | | <0.001 | | 0.682 | |  | | |
| Mc4 | Bwindi | | Western Lowland | | <0.001 | | 0.731 | |  | | |
| Mc5 | Bwindi | | Western Lowland | | 0.001 | | 0.292 | |  | | |
| Mc5 | Bwindi | | High Grauer's | | 0.001 | | 0.404 | |  | | |
| Mc5 | Virunga | | Bwindi | | 0.001 | | 0.746 | |  | | |

**Table S7.** Pairwise comparisons of residual lengths for female metacarpals across gorilla populations (log [humeral+radial length]). P-values are derived from Wilcoxon rank-sum tests with Holm correction for multiple comparisons, with significance determined at α=0.05. Only groups with ≥5 specimens per bone × elevation combination were included in analyses. Significant differences (p < 0.05) are indicated in the "Significance" column. 'High' refers to high elevation Grauer's gorillas, 'Low' to low elevation Grauer's gorillas, and 'lowland' to western lowland gorillas.

| **Bone** | | **Population 1** | | **Population 2** | **Kruskal_p** | **Holm_adjusted_p** | **Significance level** |
| --- | --- | --- | --- | --- | --- | --- | --- |
|  |  | |  |  |  |  |  |
| PP1 | | NA | | NA | NA | NA |  |
| PP2 | | NA | | NA | 0.258 | NA |  |
| PP3 | | High Grauer's | | Western Lowland | <0.001 | 0.006 | ** |
| PP3 | | Bwindi | | Western Lowland | <0.001 | 0.227 |  |
| PP3 | | Virunga | | Western Lowland | <0.001 | <0.001 | *** |
| PP3 | | Bwindi | | High Grauer's | <0.001 | 0.227 |  |
| PP3 | | Virunga | | High Grauer's | <0.001 | 0.227 |  |
| PP3 | | Virunga | | Bwindi | <0.001 | <0.001 | *** |
| PP4 | | NA | | NA | 0.399 | NA |  |
| PP5 | | NA | | NA | 0.753 | NA |  |

**Table S8.** Pairwise comparisons of residual lengths for male proximal phalanges across gorilla populations (log [humeral+radial length]). P-values are derived from Wilcoxon rank-sum tests with Holm correction for multiple comparisons, with significance determined at α=0.05. Only groups with ≥5 specimens per bone × elevation combination were included in analyses. Significant differences (p < 0.05) are indicated in the "Significance" column. 'High' refers to high elevation Grauer's gorillas, 'Low' to low elevation Grauer's gorillas, and 'lowland' to western lowland gorillas.

| **Bone** | | **Population 1** | | **Population 2** | **Kruskal_p** | | **Holm_adjusted_p** | **Significance level** |
| --- | --- | --- | --- | --- | --- | --- | --- | --- |
|  |  | |  |  |  |  | |  |
| PP1 | | NA | | NA | 0.754 | | NA |  |
| PP2 | | NA | | NA | 0.584 | | NA |  |
| PP3 | | High Grauer's | | Western Lowland | 0.027 | | 0.24 |  |
| PP3 | | Bwindi | | Western Lowland | 0.027 | | 1 |  |
| PP3 | | Virunga | | Western Lowland | 0.027 | | 0.021 | * |
| PP3 | | Bwindi | | High Grauer's | 0.027 | | 1 |  |
| PP3 | | Virunga | | High Grauer's | 0.027 | | 1 |  |
| PP3 | | Virunga | | Bwindi | 0.027 | | 1 |  |
| PP4 | | NA | | NA | 0.584 | | NA |  |
| PP5 | | NA | | NA | 0.935 | | NA |  |

**Table S9.** Pairwise comparisons of residual lengths for female proximal phalanges across gorilla populations (log [humeral+radial length]). P-values are derived from Wilcoxon rank-sum tests with Holm correction for multiple comparisons, with significance determined at α=0.05. Only groups with ≥5 specimens per bone × elevation combination were included in analyses. Significant differences (p < 0.05) are indicated in the "Significance" column. 'High' refers to high elevation Grauer's gorillas, 'Low' to low elevation Grauer's gorillas, and 'lowland' to western lowland gorillas.

| **Sex** | **Ray** | **Population 1** | **Population 2** | **Kruskal_p** | **Holm_adjusted_p** | **Significance level** |
| --- | --- | --- | --- | --- | --- | --- |
| Male | 1 | NA | NA | NA | NA |  |
| Male | 2 | NA | NA | 0.684 | NA |  |
| Male | 3 | High Grauer's | Western Lowland | 0.004 | 0.007 | ** |
| Male | 3 | Bwindi | Western Lowland | 0.004 | 0.599 |  |
| Male | 3 | Virunga | Western Lowland | 0.004 | 0.941 |  |
| Male | 3 | Bwindi | High Grauer's | 0.004 | 0.013 | * |
| Male | 3 | Virunga | High Grauer's | 0.004 | 0.013 | * |
| Male | 3 | Virunga | Bwindi | 0.004 | 0.599 |  |
| Male | 4 | NA | NA | 0.673 | NA |  |
| Male | 5 | NA | NA | 0.519 | NA |  |
| Female | 1 | NA | NA | NA | NA |  |
| Female | 2 | NA | NA | NA | NA |  |
| Female | 3 | High Grauer's | Western Lowland | 0.011 | 0.019 | * |
| Female | 3 | Bwindi | Western Lowland | 0.011 | 0.772 |  |
| Female | 3 | Virunga | Western Lowland | 0.011 | 0.772 |  |
| Female | 3 | Bwindi | High Grauer's | 0.011 | 0.039 | * |
| Female | 3 | Virunga | High Grauer's | 0.011 | 0.039 | * |
| Female | 3 | Virunga | Bwindi | 0.011 | 0.565 |  |
| Female | 4 | NA | NA | 0.273 | NA |  |
| Female | 5 | NA | NA | 0.223 | NA |  |

**Table S10**. Kruskal–Wallis and pairwise Wilcoxon test results (Holm-adjusted) comparing residuals from RMA regressions of log-transformed proximal phalanx length on metacarpal length across gorilla populations.

| **Sex** | **Elevation** | **Mean Residual** | **Median Residual** | **n** |
| --- | --- | --- | --- | --- |
| Male | Low Grauer's | 0.063 | 0.076 | 3 |
| Male | High Grauer's | 0.054 | 0.063 | 11 |
| Male | Virunga | <0.001 | <0.001 | 21 |
| Male | Western Lowland | <0.001 | <0.001 | 26 |
| Male | Bwindi | <0.001 | <0.001 | 12 |
| Female | High Grauer's | 0.049 | 0.035 | 8 |
| Female | Virunga | <0.001 | 0.009 | 19 |
| Female | Western Lowland | <0.001 | 0.002 | 23 |
| Female | Bwindi | <0.001 | <0.001 | 7 |

**Table S11.** Mean and median residuals from RMA regressions of log-transformed proximal phalanx length on metacarpal length (PP3 ~ Mc3) by population and sex. Negative residuals indicate proportionally shorter proximal phalanges relative to metacarpal length.

**Figure S1a.** **Allometric scaling of gorilla metacarpals (Log[metacarpal length] vs. Log[Body Mass])**

**Figure S1b. Allometric scaling of gorilla proximal phalanges (Log[proximal phalangeal length] vs. Log[Body Mass])**
